# Structural and Chemical Modifications Towards High-Performance of Triboelectric Nanogenerators

**DOI:** 10.1186/s11671-021-03578-z

**Published:** 2021-07-30

**Authors:** Yerzhan Nurmakanov, Gulnur Kalimuldina, Galymzhan Nauryzbayev, Desmond Adair, Zhumabay Bakenov

**Affiliations:** 1 School of Engineering and Digital Sciences, Nazarbayev University, Kabanbay Batyr Ave. 53, Nur-Sultan, 010000 Kazakhstan; 2 Department of Mechanical and Aerospace Engineering, School of Engineering and Digital Sciences, Nazarbayev University, Kabanbay Batyr Ave. 53, Nur-Sultan, 010000 Kazakhstan; 3 Department of Electrical and Computer Engineering, School of Engineering and Digital Sciences, Nazarbayev University, Kabanbay Batyr Ave. 53, Nur-Sultan, 010000 Kazakhstan; 4 Department of Chemical and Materials Engineering, School of Engineering and Digital Sciences, Nazarbayev University, Kabanbay Batyr Ave. 53, Nur-Sultan, 010000 Kazakhstan

**Keywords:** Triboelectric nanogenerator, Surface micro-/nano-patterning, Chemical functionalization, Wearable electronics, Self-charging power systems

## Abstract

**Abstract:**

Harvesting abundant mechanical energy has been considered one of the promising technologies for developing autonomous self-powered active sensors, power units, and Internet-of-Things devices. Among various energy harvesting technologies, the triboelectric harvesters based on contact electrification have recently attracted much attention because of their advantages such as high performance, light weight, and simple design. Since the first triboelectric energy-harvesting device was reported, the continuous investigations for improving the output power have been carried out. This review article covers various methods proposed for the performance enhancement of triboelectric nanogenerators (TENGs), such as a triboelectric material selection, surface modification through the introduction of micro-/nano-patterns, and surface chemical functionalization, injecting charges, and their trapping. The main purpose of this work is to highlight and summarize recent advancements towards enhancing the TENG technology performance through implementing different approaches along with their potential applications.

**Graphic Abstract:**

This paper presents a comprehensive review of the TENG technology and its factors affecting the output power as material selection, surface physical and chemical modification, charge injection, and trapping techniques.
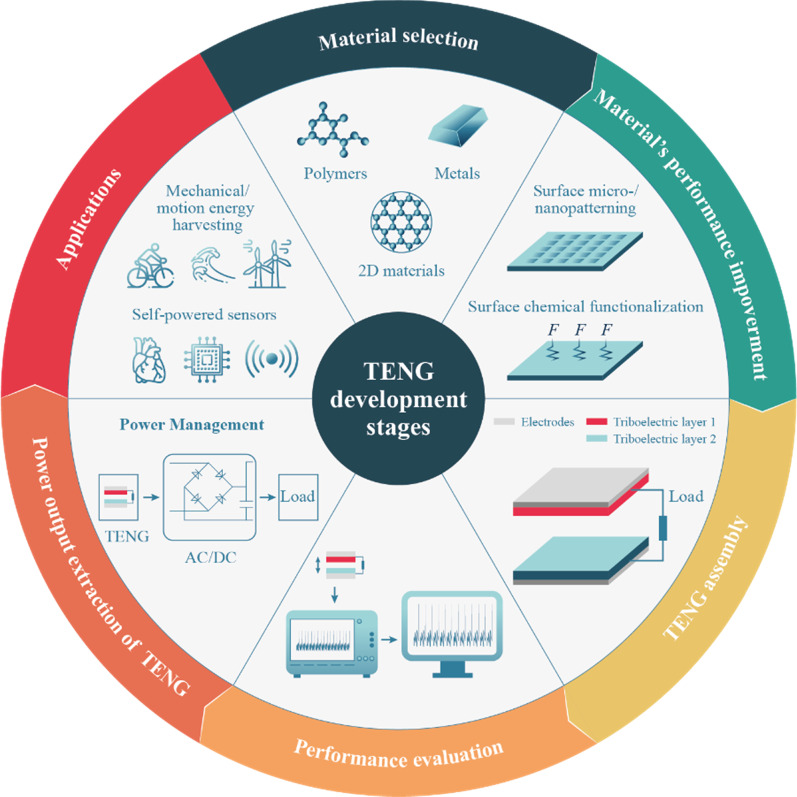

## Introduction

Currently, intense work is dedicated to realizing alternative ways of producing electricity to meet the ongoing demands of more sustainable and environmentally friendly power sources. A promising solution is to harvest abundant ambient energy from the environment, where it is represented in the form of solar energy [1], thermal energy [2], wind energy [3], and mechanical energy [4]. However, the reliable operation of solar cells, thermoelectric generators and wind turbines relies on specific ambient conditions, such as the constant availability of sunlight, temperature, and wind power [5]. Therefore, developing more environmentally independent and active energy acquisition methods is of paramount significance. Recent advances in Internet-of-Things (IoT), cloud computing, personal electronics, and artificial intelligence have emerged a vast number of user-oriented functional wearable electronics. Thus the necessity for light and sustainable power sources is of high importance [6–8].

Moreover, apart from being lightweight and sustainable, these power sources should also be flexible, stretchable, and eco-friendly. Mechanical energy harvesting is an attractive way of creating fully integrated and wearable self-powered microsystems that overcome the drawbacks of capacitors/batteries such as limited capacity, short operational life, high charging frequency, and potential safety risks [9]. Recently, the technologies of harvesting electricity at micro/nanoscale have attracted a lot of attention. For instance, electromagnetic microgenerators (EMGs) [10], piezoelectric nanogenerators (PENGs) [11], and triboelectric nanogenerators (TENGs) [12] are of great interest. Among them, TENG has proven to be a powerful technology for generating electricity from low-frequency mechanical energy. TENG was first reported by Wang and co-workers [13] in 2012, and subsequent studies in this area highlighted the advantages as high-output performance, lightweight, low cost, available material selection, simple design, and environmental friendliness [8, 14]. Since the first report, promising results have been achieved in the development of TENG with instantaneous peak output power density reaching 500 W/m^2^ and with an instantaneous power conversion efficiency of 85% [15].

Furthermore, output voltage or current profiles of TENG can be applied as active sensing signals for the detection of mechanical or even chemical stimuli exerted on them. The electrical output of TENG is constantly improving through surface modification (chemical and physical), fabrication process optimization and device design. The generation of static polarized charges is related to contact electrification followed by electrostatic induction, where mechanical energy is converted into electrical energy. Therefore, enhancement of the charge generation during contact-electrification is one of the most important factors for further developing and improving TENG technology.

Considering the discussion above, this paper presents a comprehensive review of TENG and its factors affecting the output power, e.g., material selection, surface physical and chemical modification, charge injection, and trapping techniques. The possibility of physical hybridization of TENG with the other energy harvesting technologies and suggested applications for TENG have also been summarized. This review will guide researchers on the methods and techniques that could motivate further investigations and develop higher output TENGs in the future.

## Fundamentals and Materials for TENGs Fabrication

### Triboelectrification Mechanism

The general working principle of TENG is based on the contact electrification and electrostatic induction of two different materials, where these materials become electrically charged after getting in close contact with each other [16, 17]. An injection of charges occurs from one material to another when two material surfaces are brought into contact, generating an equal amount of positive and negative surface charges. If the materials are then separated, each surface will hold either the positive or negative charges while being isolated by a gap. The sign of the charges carried by a particular material depends on its relative polarity compared to the other material with which it was in contact. A small current will flow to balance the charges through an electrical load connected to two electrodes placed at the back of the two surfaces. An alternating current (AC) is produced by continuously repeating the contact-separation process [18].

**Fig. 1:**
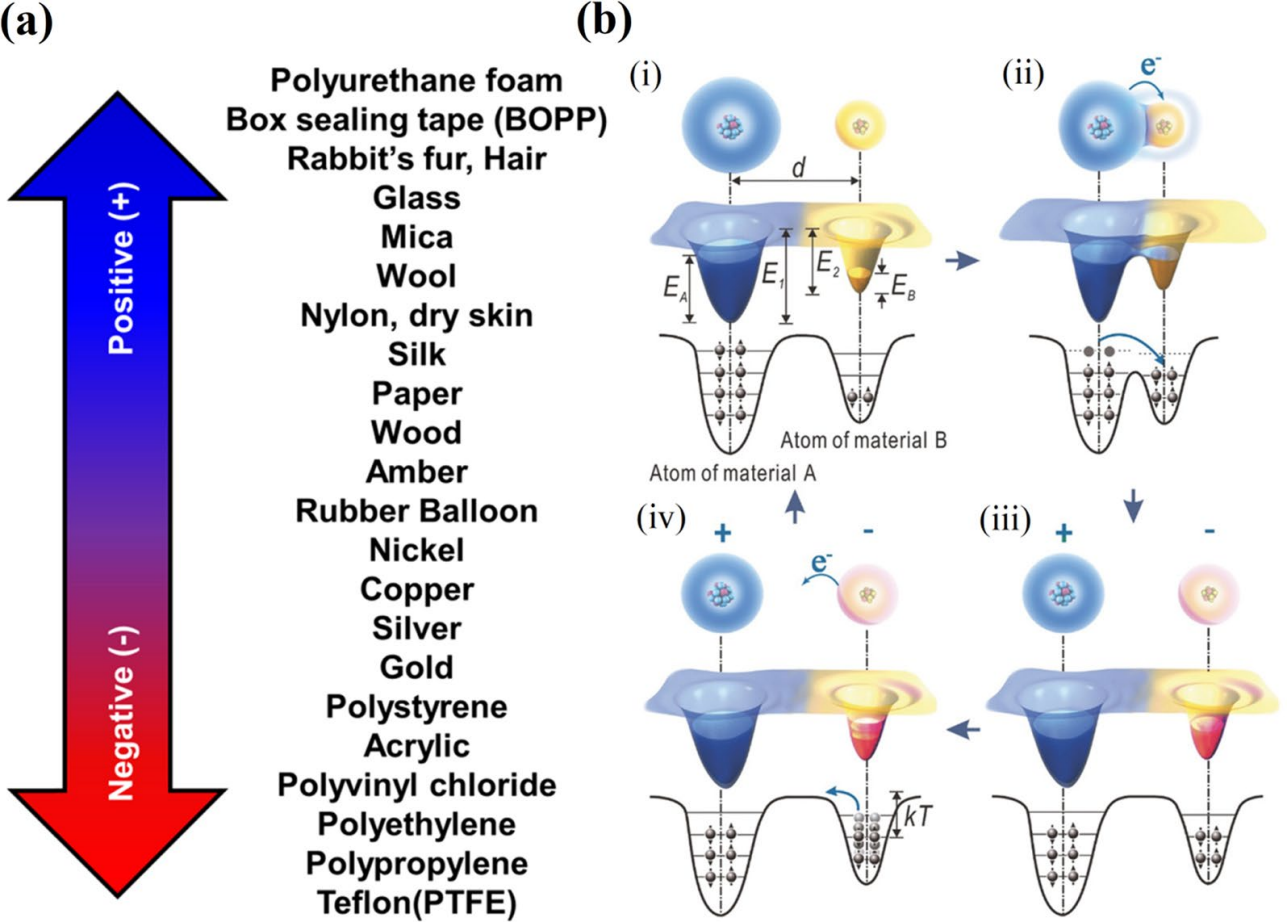
**a** Schematic of triboelectric materials in series depending on their relative triboelectric charge attracting/receiving characteristics (Reproduced with permission from Ref [19].). **b** The atomic-scale-electron-cloud-potential-well model to describe the contact electrification process of a TENG (Reproduced with permission from Ref [21].)

Materials are ranked according to their tendency to acquire electrons or lose electrons in triboelectric series (Fig. 1a), and the position of the material determines the efficiency of charge exchange [19], and by the selection of triboelectric pair charge density and output characteristics of the TENG can be predicted.

The triboelectrification mechanism is still not fully understood, and it is supposed that a chemical bond, called adhesion, is formed between two surfaces during the contact. As a result, charges move from one material to another to balance their electrochemical potential [20]. Recently, several models were reported to describe the contact electrification process. For instance, Xu et al*.* proposed [21] an atomic-scale-electron-cloud-potential-well model, and according to their model, when atoms of two materials are far apart from each other, their electron clouds are not overlapping and their electrons are tightly held on their original orbits with a high escaping energy barrier (Fig. 1b). However, when mechanical stimuli are applied to bring materials closer to each other, their atoms get into the area of the interatomic repulsive region an overlap of their electron clouds takes place, leading to the drastic reduction of the interatomic potential barrier. Consequently, high-energy electrons of one material can easily overcome the reduced barrier and flow to the second material. As a result, some electrons in the first material with higher energy will have reduced potential barrier and transfer to the second material to maintain equilibrium.

There are other ways of interpreting the surface charging process through various parameters as the electron affinity [22], work function [23], outermost element [24], and internal polarity [25] of the two materials [19]. It should be noted that two identical surfaces can also generate triboelectricity when they are brought in contact [26].

**Fig. 2:**
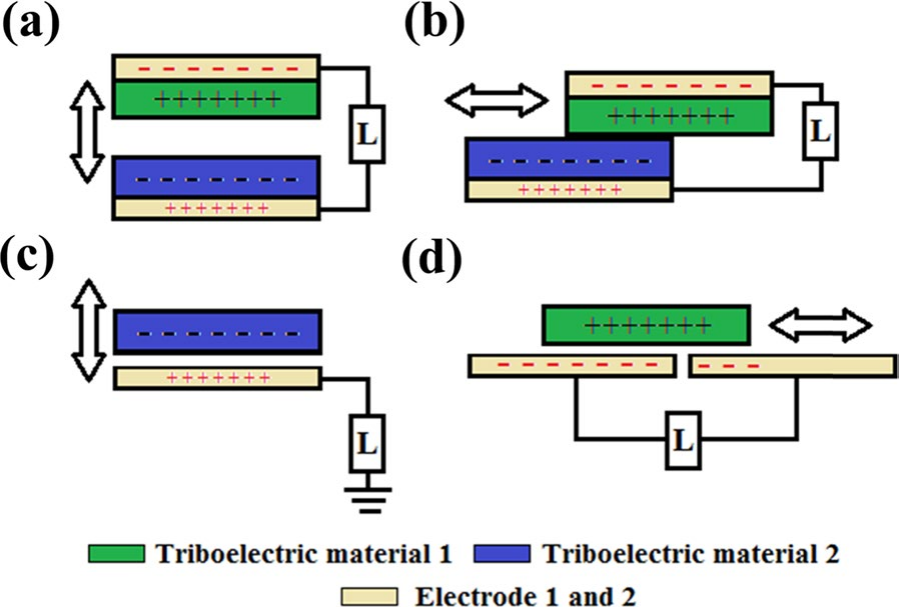
The four fundamental operation modes of TENG: **a** Vertical contact-separation mode (VCSTENG), **b** lateral sliding mode (LSTENG), **c** single-electrode mode (SETENG) and **d** freestanding triboelectric-layer mode (FSTENG)

Over the past years, TENGs of various architectures and designs have been fabricated for energy harvesting and sensing. Their main operation principles can be divided into four modes (Fig. 2) which are: a vertical contact-separation mode (VCSTENG), a lateral sliding mode (LSTENG), a single electrode mode (SETENG), and a free-standing triboelectric layer mode (FSTENG) [27–29].

### Material for TENG Fabrication

All materials, regardless of nature, have a triboelectrification effect and can be used for TENG fabrication. However, the performance of a TENG is directly related to a proper material selection because the materials should easily generate charges with opposite triboelectric polarities. The density of such charges influences both output voltage and current due to the superposition principle of the electric potential [30]. So far, the most popular materials applied in TENG fabrication are natural and synthetic polymers, metals and alloys, and two-dimensional (2D) materials like graphene and its derivatives, MXenes.

#### Polymers as a Material for TENG Fabrication

Natural and synthetic polymers are widely represented in the triboelectric series and are among the first choices for TENG devices due to their excellent dielectric properties and mechanical robustness. Moreover, they demonstrate consistent charge transfer patterns related to the chemical nature of specific surfaces [31]. Although we can fabricate TENG from a randomly chosen triboelectric pair, not every pair can demonstrate a high output performance. The TENG performance is highly dependent on the electron affinity difference of the triboelectric material pairs. Therefore, the polymeric materials that contain fluorine, such as polytetrafluoroethylene (PTFE), fluorinated ethylene propylene (FEP), and polyvinylidene fluoride (PVDF), are always the first candidates to be used as electron-accepting layers in TENG due to the strong electron attractive force of a fluorine element. On the other hand, polymers, such as nylon, thermoplastic polyurethane (TPU), melamine formaldehyde (MF), and polypyrrole (PPy), are mainly used as electron-donating layers [32]. Thus, based on the analyses of chemical structures of the polymers, one can observe that nitrogen-containing polymers with pyridine, amine and amide groups develop the most positive charges. In contrast, halogenated (*e.g.*, fluoride and chloride) polymers form the most negative charges [25].

**Fig. 3:**
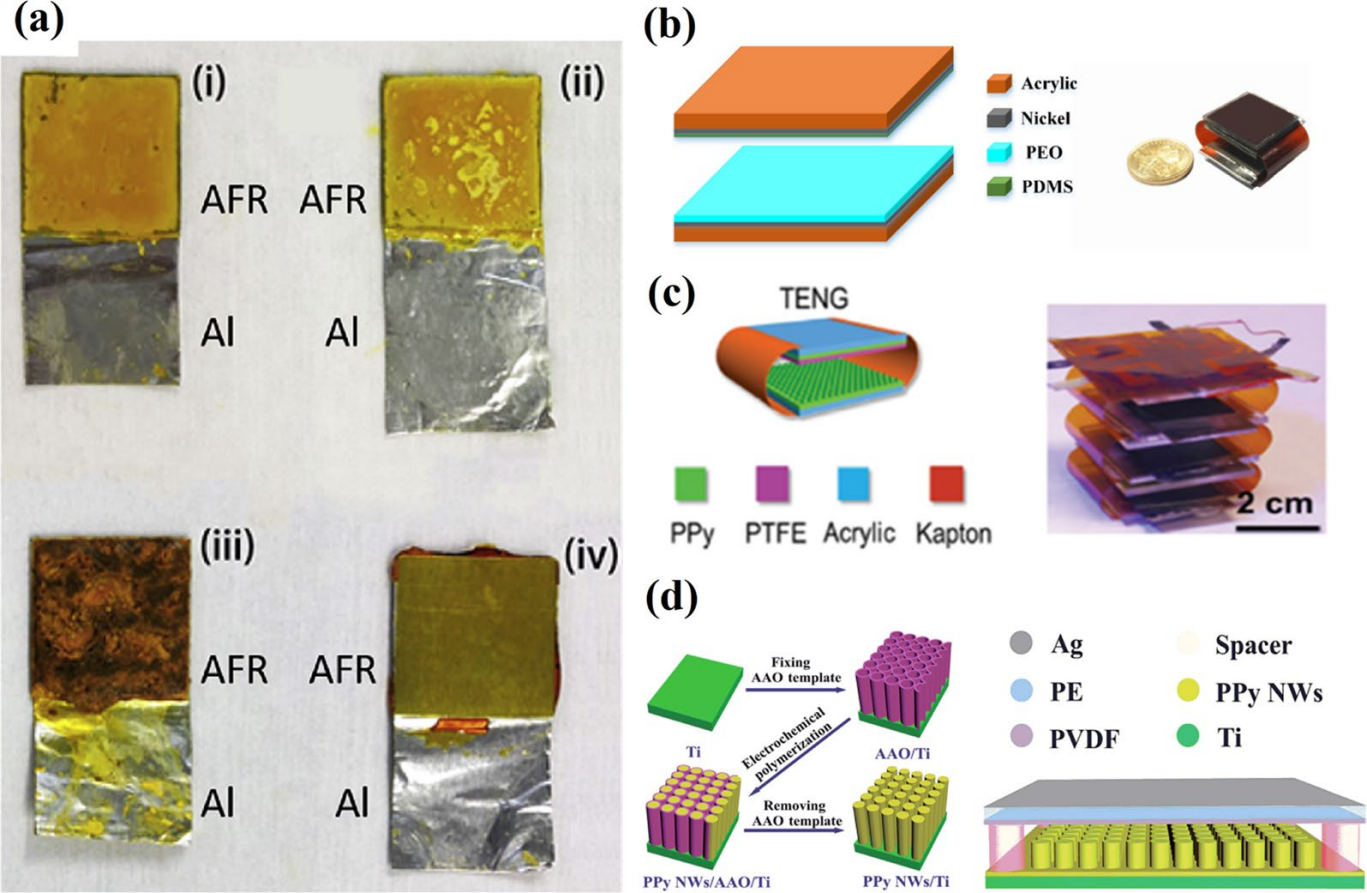
Tribo-positive polymers for the TENG fabrication: **a** the digital photographs of the AFR sample at various stages of preparation with (i) corresponding to as-prepared uncured sample deposited on an Al foil, (ii) corresponds to washed sample while (iii) and (iv) correspond to the dried and fully cured, hot-pressed AFR samples, respectively (Reproduced with permission from Ref. [31]), **b** a schematic diagram and a photo of the TENG using PEO and PDMS films as the positive and negative tribo-materials, respectively (Reproduced with permission from Ref. [33]), **c** a schematic diagram and a photo of three stacked PPy-based TENG (Reproduced with permission from Ref. [34]), d a schematic depiction of PPy NW preparation by electrochemical polymerization with AAO as the template and schematic diagram of the final TENG. (Reproduced with permission from Ref. [35])

It is pertinent to note that the choice of tribo-negative materials is quite wide compared to that of the tribo-positive materials. The choice of materials is equally crucial for improving the surface charge density and subsequently generated power in TENG. Recently, many studies on the creation of tribo-positive materials have been focused on using polymeric materials, both artificial and natural. Zhao et al*.* [31] reported aniline formaldehyde resin (AFR) films as a tribo-positive material to fabricate high-performance TENG combined with PTFE. These films were obtained during the condensation in an acidic medium between aniline and formaldehyde. The process resulted in abundant nitrogen and oxygen surface functional groups that can acquire a tribo-positive charge (Fig. 3a). The peak-to-peak voltage and current density of the thin-layered AFR-based TENG were about 1000 V and 65 mA/m^2^, respectively. The application of pristine poly(ethylene oxide) (PEO) films as the positive tribo-material to fabricate high-performance TENG in combination with polydimethylsiloxane (PDMS) has also been reported (Fig. 3b) [33]. A simple spin-coating process-based PEO/PDMS TENG (without any complicated micro/nano-patterning) produced a peak-to-peak voltage of 970 V, a current density of 85 mA/m^2^, and a peak power density of about 40 W/m^2^. On the other hand, Wang et al*.* [34] utilized a micro/nanostructured PPy film with a hollow horn-like surface morphology as a tribo-positive layer to fabricate a multi-layered all-plastic-materials-based TENG (Fig. 3c). The maximum output power of 5.5 W/m^2^ was generated by a three-parallel TENG, which was able to light 32 LEDs by manual pressing only.

Another TENG, based on the PPy nanowires (NWs) material as an electron donor, was obtained by an electrochemical polymerization method with anodic aluminum oxide (AAO) as the template (Fig. 3d) [35]. The PPy NWs structure with a diameter of 100 nm was developed to increase the frictional contact area, and the obtained high output performance TENG showed a maximum short circuit current density (*Jsc*) of 23.4 mA/m^2^ and output voltage of 351 V, with an ability to light 372 commercial LEDs.

**Fig. 4:**
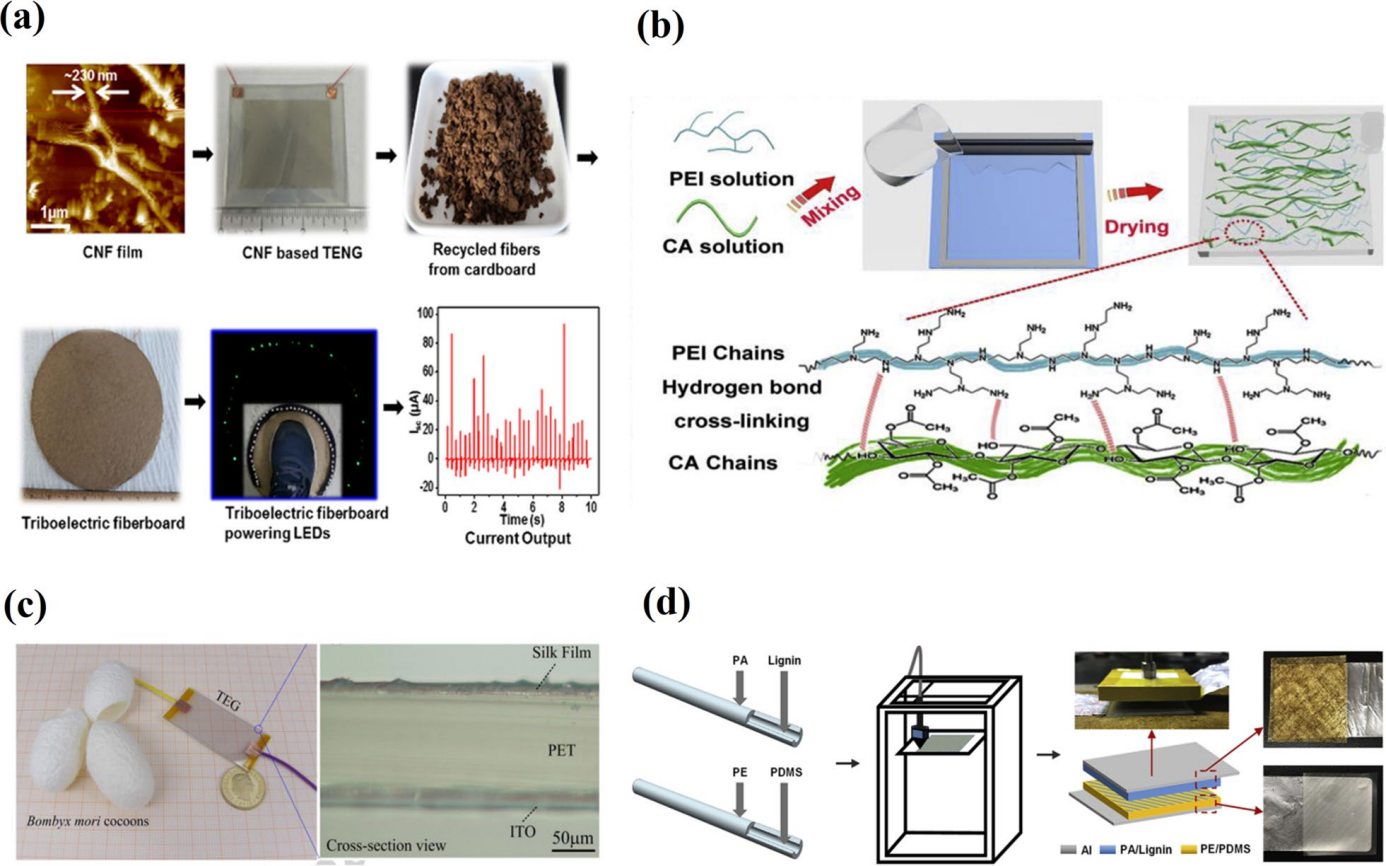
Fabrication of TENGs from eco-friendly materials: **a** CNF-based TENG. (Reproduced with permission from Ref. [37]); **b** a schematic of the fabrication process for CP biocomposite and interactions between the CA and PEI molecular chains. (Reproduced with permission from Ref. [38]); **c** a photo of the silk-based TENG and cross-section microscope image of its bottom layer. (Reproduced with permission from Ref. [39]); d the process for preparing IBTENG and the photos of friction layers and fabricated IBTENG. (Reproduced with permission from Ref. [40])

Conventional materials used for the TENG device preparation are associated with heavy metal and plastic pollution of soil. Recently many studies have aimed at the development of eco-friendly material applications [36]. Yao et al*.* [37] reported the implementation of biodegradable and abundant cellulose nanofibrils (CNFs) in the TENG devices. These flexible and transparent CNFs are stated as being excellent tribo-positive material. CNF was paired with FEP for further integration with a fiberboard made from recycled cardboard fibers using a chemical-free cold pressing method (Fig. 4a). The device showed good electric outputs (i.e., ~ 30 V and ~ 90 μA), when the force of a human step was applied.

In another work [38], a highly flexible, porous electroactive biocomposite tribo-negative material has been proposed on a base of cellulose acetate (CA). For that purpose, CA-polyethyleneimine (PEI) biocomposite (CP biocomposite) has been paired with a flexible low-temperature vulcanized silicone rubber (LTV) as a tribo-negative membrane to develop the high-output CP/LTV-TENG. A simple polymer-blended method was used to prepare the CP biocomposite. The thin CP biocomposite (Fig. 4b) was obtained by drying the acrylic groove model, coated by the resultant homogeneous mixture of CA and PEI solutions.

Another excellent tribo-positive material, namely, silk fibroin for TENG preparation (Fig. 4c), was introduced by Zhang et al*.* [39]. The investigated biodegradable material was suggested as promising due to its high-output performance, with a maximum voltage of 268 V, current of 5.78 μA, and power density of 193.6 μW/cm^2^ measured. Insoluble and infusible biomass-based TENG (IBTENG) was fabricated, where the biomass materials (*i.e.*, lignin, chitosan and cellulose) and PDMS were used as tribo-positive and tribo-negative materials, respectively [40]. The IBTENG friction layers were prepared as, first, the biomass materials, such as lignin or chitosan (CTS) and PDMS, were separately poured into the polyamide (PA) and polyethylene (PE) tubes with an inner diameter of 1 mm. After, they were molded into different friction layers (PA/CTS, PA/Lignin, or PE/PDMS) by fused deposition modeling (FDM) (Fig. 4d). The mechanical energy conversion efficiency of 78% was reached with the output power measured at 308 V times 61.6 μA at a frequency of 30 Hz. Besides, the other naturally-derived biopolymers, *e.g.*, starch polymer [41], hydrogels [42], peptide, and NWs [43], have been developed and applied in high-performance TENG fabrication.

**Fig. 5:**
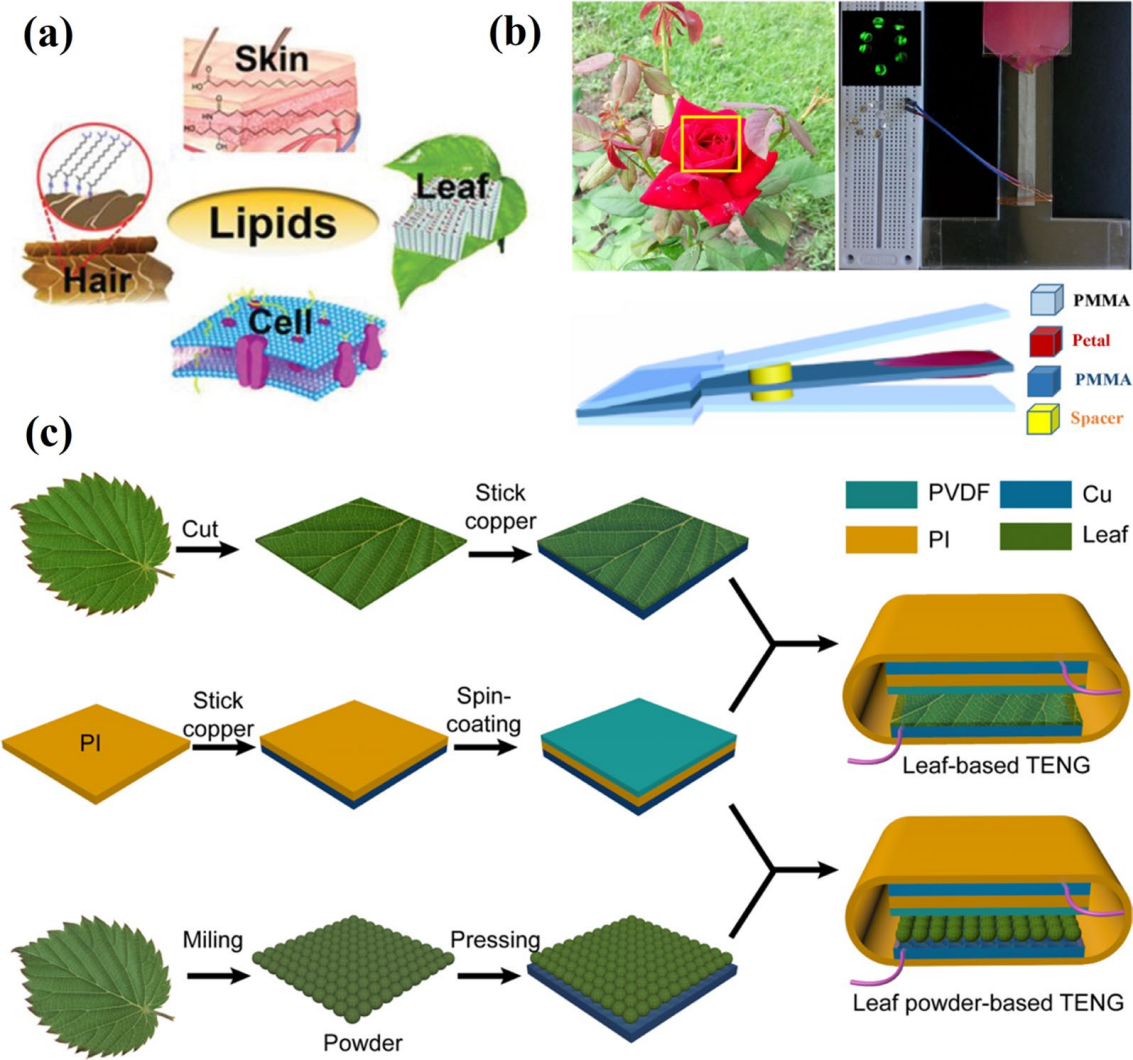
TENGs based on regenerative natural substances: **a** lipid layers on natural regenerative surfaces. (Reproduced with permission from Ref. [44]); **b** a preparation of the biodegradable plant leaf and leaf powder-based TENGs. (Reproduced with permission from Ref. [45]); **c** TENG based on Chinese red rose. (Reproduced with permission from Ref. [46])

Positive triboelectricity properties of regenerative natural substances (*e.g.*, skin, hair, etc.) have attracted attention, and studies related to investigating their high tribo-positivity have been carried out. It has been reported that the lipid layer responsible for high positive triboelectricity opens up the possibility of energy harvesting without causing any damage or change to the environment (Fig. 5a) [44]. A biodegradable plant leaf and leaf powder-based TENGs were fabricated through simple and cost-effective methods [45]. The TENG device prepared from the dried leaf powder was obtained by applying it uniformly onto Cu tape, followed by attaching it to the backside of a Cu tape with a silver paste (Fig. 5b). PVDF was used as the negative part of TENG.

Moreover, the poly-L-Lysine modified fresh leaf and leaf powder-based TENGs were also prepared and had improved output performance. Among them, after the surface modification, the poly-L-Lysine modified leaf powder-based TENG demonstrated superior performance (with a short-circuit current (*I*_*sc*_) of 60 μA and open-circuit voltage (*V*_*oc*_) of 1000 V) and was able to charge a commercial electric watch or 868 LEDs. The TENG based on petal (Chinese rose) and poly(methyl methacrylate) (PMMA) gave a *V*_*oc*_ of 30.6 V and *I*_*sc*_ of 0.78 μA under a constant periodical compressive force (100 N) at a frequency of 2 Hz (Fig. 5c) [46]. The surface of the petals rich in micro-and nanostructures provides a sufficient roughness for hydrophobicity, suggesting the practical generation of energy from raindrops. The measured *I*_*sc*_ generated from the petal with falling water droplets on its surface was equal to 7.84 nA.

#### Application of 2D Materials for TENG Fabrication

**Fig. 6:**
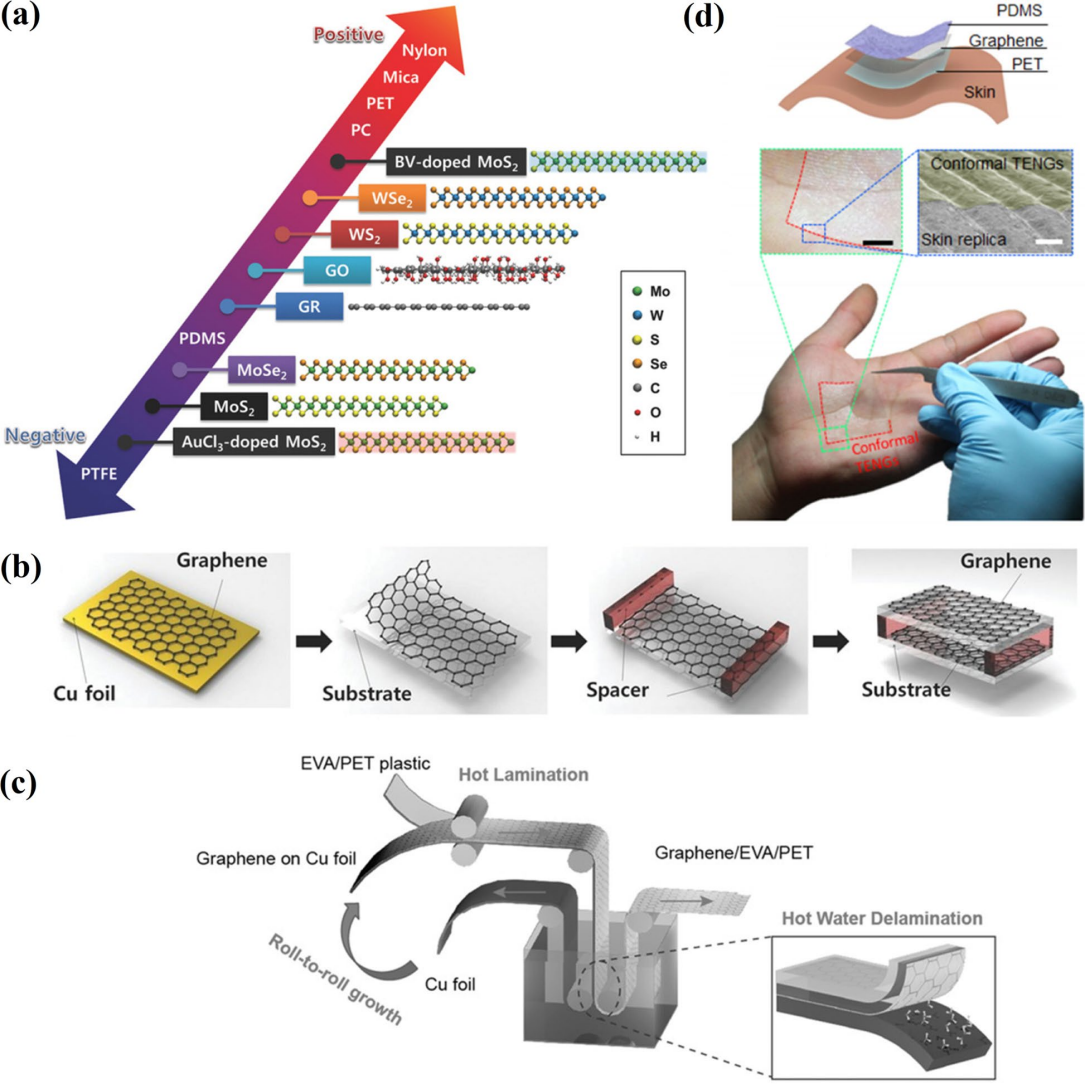
Applications of 2D materials in TENG fabrication: **a** modified triboelectric series including 2D materials (Reproduced with permission from Ref. [48]); **b** a schematic of fabrication of 1L graphene-based TENG (Reproduced with permission from Ref. [52]); **c** a schematic of the roll-to-roll delamination of Cu and graphene onto EVA/PET substrate (Reproduced with permission from Ref. [53]); **d** a schematic of the conformal TENGs and images of the conformal TENGs attached on the palm (Reproduced with permission from Ref. [54])

2D materials are one of the most studied materials due to their unique properties and applicability in various fields, including energy harvesting [47]. Seol et al. [48] investigated the triboelectric behavior of different 2D-layered materials, *e.g.*, MoS_2_, MoSe_2_, WS_2_, WSe_2_, graphene, and graphene oxide (GO) (Fig. 6a). The ability of graphene to store an electric charge for an extended time has been revealed during the theoretical studies, suggesting its suitability for TENGs [49, 50]. The graphene performance can be improved by structure modification, which is reached by chemical vapor deposition (CVD), introducing wrinkles and ripples. A significant friction and surface charge created by the triboelectric effect makes it more suitable for high output voltage applications [51]. The possibility of generating electrical energy from mechanical stress using graphene was demonstrated by Kim et al. [52]. The authors reported that CVD-grown graphene could serve as an efficient frictional material in TENGs. Several types of nanogenerators (NGs) were fabricated by variating the grown graphene layers on Cu and Ni foils. The one-layer (1L), two-layer (2L), three-layer (3L), four-layer (4L), and few-layer graphene layers were obtained (Fig. 6b). Here, 1L graphene was grown on a Cu foil using CVD, followed by a wet transfer method on a flexible PET substrate. The PET/1L graphene (bottom side) substrate and the PET/graphene (top electrode) substrate were assembled into the TENG using a plastic spacer between the bottom and upper parts. The graphene-based TENG showed reasonable flexibility, stretch-ability and adjustability, and the simulation results demonstrated an appropriate potential distribution. The prepared 1L graphene demonstrated a high-output voltage of 5 V and a current density of 500 nA/cm^2^.

Moreover, TENG fabricated by ordinary stacked few-layer graphene exhibited enhanced output voltage and output current density of 9 V and 1.2 mA/cm^2^. The effects of work function and friction were the main factors in comparing the TENG performance and depending on the number of graphene layers. Chandrashekar et al*.* [53] reported the fabrication of transparent TENG by the CVD method. The graphene was transferred by a roll-to-roll and etching-free method from Cu to ethylene vinyl acetate/poly(ethylene terephthalate) (EVA/PET) plastic using surface-energy-assisted delamination in the hot deionized water (Fig. 6c). Wearable transparent and flexible graphene/EVA/PET film-based TENG demonstrated a maximum output voltage and current density of 22 V and 0.075 µA/cm^2^, respectively.

TENGs directly applied to the human skin and operating as part of wearable devices were fabricated based on thin graphene (< 1 nm) (Fig. 6d) [54]. The triboelectric electricity generated on human skin during contact with various clothes was applied as self-powered touch sensors. Kwak et al*.* [55] reported a promising power output from a single moving water droplet on monolayer graphene and PTFE, which had a value of 1.9 mW. Monolayer graphene was grown on *Cu* foil using CVD at 1000 °C, applying CH_4_ and H_2_ as the reaction mixture gas. Then PMMA was spin-coated on the graphene/Cu foil, and the PMMA/graphene/Cu foil was dipped into an etchant (HNO_3_) solution for Cu etching. Subsequently, PMMA/graphene was transferred onto a PTFE substrate after removing the PMMA in an acetone solution.

**Fig. 7:**
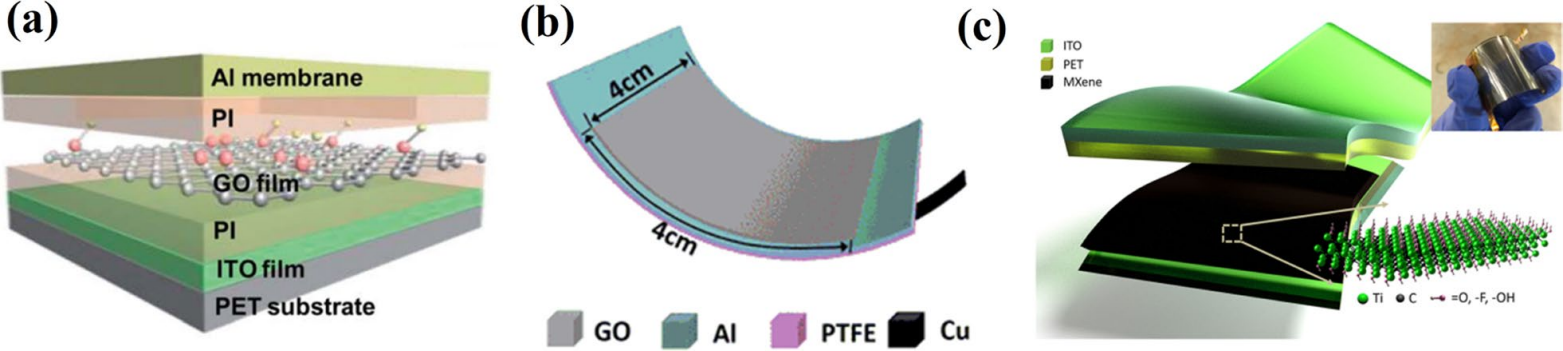
Applications of 2D materials in TENG fabrication: **a** a multilayer structure of the Al/PI/GO/PI/ITO/PET TENG (Reproduced with permission from Ref. [57]); **b** a structure of the designed S-TENG (Reproduced with permission from Ref. [58]); **c** a structure of the MXene-based TENG (Reproduced with permission from Ref. [60])

GO has been attracting attention for high-performance TENG output similar to graphene due to its high specific surface area [56]. For instance, Tian et al*.* [57] reported a flexible electrostatic NG based on GO film combined under a multilayer structure of Al/PI/GO/PI/ITO on a flexible PET substrate (Fig. 7a). Such GO-based TENG demonstrated a high-output power of 60 nW at 15 N force at 1 Hz. In another work, Guo et al*.* [58] also used GO in the SETENG, where the GO film is stacked on an Al electrode connected with a load resistor (Fig. 7b). A stable TENG performance was obtained by fixing the Al electrode onto a PTFE substrate. This device was found to generate electricity with a high power density of 3.13 W/m^2^ from ambient movements and detect the dynamic force with an outstanding sensitivity close to 388 μA/MPa. Such parameters of the obtained TENG could be suitable for running self-powered sensors and even portable/wearable electronics.

Recently, MXenes (transition metal carbides, nitrides) have also shown their high conductivity and unique structure as 2D-layered additives in energy generating or storing materials. MXenes is a family of about 30 synthesized material compositions with a wide range of electrical and mechanical properties adjusted by tuning the composition and surface functional groups [59]. Importantly, Dong et al*.* [60] demonstrated that 2D titanium carbide MXenes (Ti_3_C_2_T_x_, where T_x_ are surface functional groups such as –O, –OH, and –F) are triboelectrically more negative than PTFE (Teflon). The reported MXenes-based TENG (Fig. 7c) can exhibit both high *V*_*oc*_ (varying from ~ 500 to ~ 650 V) and instantaneous peak power (up to ~ 0.5–0.65 mW). Such characteristics can power more than 60 LEDs or quickly charge a 1 µF capacitor up to 50 V. Moreover, these devices can generate electrical power from simple muscle movements even when the device is flexed by about 30°, which is very typical for integration with wearable electronics.

As was discussed earlier, the choice of materials for TENG development is broad and potentially can extend further with the development in the field. However, selecting the proper materials pair is not the only necessary process of improving TENG performance. Further modifications (physical and chemical) are required to be applied for the surface, morphology, and charge generation improvements.

### Figure of Merit (FOM) for TENG

The number of applicable materials for TENG fabrication and its various working modes has caused difficulties in measuring and evaluating the performance of different TENGs. A specialized figure of merit (*FOM*) for TENG in which a performance *FOM* (*FOM*_*P*_) for TENG consisting of a structural *FOM* (*FOM*_*S*_) and a material *FOM* (*FOM*_*M*_) was suggested [61].

The *FOM*_*P*_ proposed as a standard by Zi et al*.* [61] to normalize the parameters and evaluate quantitatively TENG, which considers materials, structures, surface charge densities, contact area and distance between contacting surfaces.

The structural *FOM* (*FOM*_*S*_) that depends only on structural characteristics is given by the following equation.

1 $$FOMs = \frac{{2{\upvarepsilon }_{0} }}{{\delta^{2} }}\frac{{E_{m} }}{{Ax_{max} }}$$

*E*_*m*_ is the largest possible output energy per cycle, *x*_*max*_ is the maximum displacement of tribo-pair, *δ* is charge density, *ε*_*0*_ is the permittivity of vacuum and *A* is the contact area.

Consequently, the expression for calculating *FOMp* is.

2 $$FOMp = FOM_{s} \cdot \delta^{2} = 2{\upvarepsilon }_{0} \frac{{E_{m} }}{{Ax_{max} }}$$

where a material *FOM*_*M*_ is defined as *δ*^*2*^.

In brief, *FOM*_*P*_ can be used as the common standard to evaluate different TENGs, because it represents the maximum possible output power and closely related to the highest achievable energy-conversion efficiency; also, *FOM*_*P*_ is universally applicable for TENG systems with various device structures, working modes, used materials, and sizes.

## Enhancement of triboelectric charge density

### Surface Micro-/Nano-Structuring

The performance of TENG is directly related to the charge density on the contact surface, therefore, enhancing the charge generation is the main approach for better performance [16]. The charge density squared is the main parameter used to quantify the TENG performance as a material figure of merit [62]. So far, several strategies to increase the output electric power have been reported, including the fabrication of microscale or nanoscale surface structures to raise the contact area [14] and surface chemical modification [63] to increase the surface charge density or to facilitate the triboelectric charge transfer. The overall number of generated charges can be improved by directly enlarging a contact surface area. Especially, introducing morphologies like NWs [64], nanoparticles [65], or other nanoscale patterns are intensively applied to improve the surface area of triboelectric materials to increase charge-carrying sites.

**Fig. 8:**
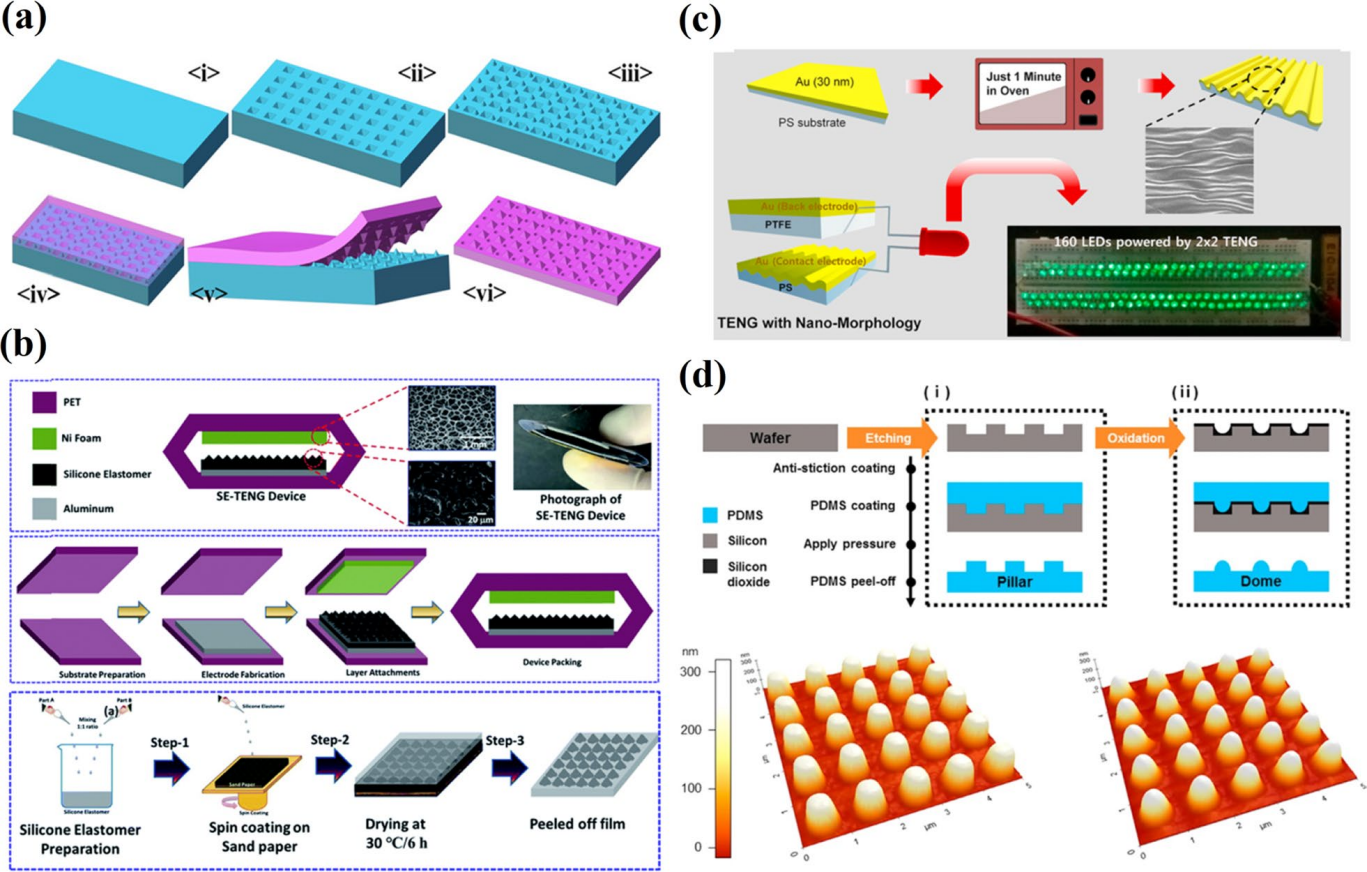
Surface micro-/nano-patterning techniques: **a** a process flow of soft lithography (Reproduced with permission from Ref. [67]); **b** SE-TENG device schematic and fabrication (Reproduced with permission from Ref. [68]); **c** a process of the glass transition of the PS for obtaining nano-to-micro scale morphology (Reproduced with permission from Ref. [69]); **d** an illustration of the fabrication of the dome-shaped and pillar-shaped nanostructures by the replica molding process on the silicon wafers, and their surface taken by an atomic force microscope (Reproduced with permission from Ref. [70])

Moreover, a soft lithography technique for fabricating or replicating microstructures using elastomeric stamps/molds [66], has found a potential application in preparing energy harvesting devices. The example of a soft lithography application was shown in Fig. 8a, where soft liquid or solid material is used to cover a pre-patterned stamp or mold. Then the solidification of the soft material takes place under a specific external treatment (e.g., radiation, heating). Lastly, the micro-patterned solid-phase soft material is separated from the stamp or mold [67].

The cost-effective soft lithography technique was applied to create micro-roughness on the silicone elastomer film as an active layer in SETENG fabrication. Its step-by-step fabrication is illustrated in Fig. 8b. The three SETENG devices were fabricated using this silicone elastomer as the negative triboelectric layer and Al, Cu and Ni foam as the positive electrodes. The output was higher in the TENG device made of Ni foam due to its porous nature than Al and Cu. The highest voltage and current values of SETENG were 370 V and 6.1 μA, respectively, with a maximum area power density of 17 mW/m^2^ at a load of 1 GΩ resistance. To show the durability of the SETENG device, a stability test was carried out for 2000s. During the test, the device demonstrated a uniform peak pattern (every 600 s) from the start of the test until 2000s. As the authors claim, this shows that the device can work for an extended time with a stable electrical output [68].

In other research by Kim et al*.* [69], a novel approach to improve the output power of polystyrene (PS)-based TENG by glass transition of PS was demonstrated. The authors introduced a preparation of a nano-to-micro morphology without using high-level equipment by using only a heating process in the air for 1 min. Here, gold with a thickness of 30 nm was deposited onto a PS film to a thickness of 200 µm via a thermal evaporation method (Fig. 8c). Tcho et al. [70] reported PDMS with well-ordered protruding nanostructures, with pillar and dome shapes, using a physical modification of the surface morphology (Fig. 8d). These well-ordered dome-shaped and pillar-shaped nanostructures, with accurately controlled identical heights and diameters (245 nm and 500 nm, respectively), were created on the PDMS film by a replica molding process.

Further, the TENG properties such as force sensitivity and durability were investigated in terms of surface morphology geometric shape effects. The investigations revealed that TENG with dome-shaped PDMS (DP-TENG) showed greater force sensitivity than TENG with pillar-shaped PDMS (PP-TENG). On the contrary, based on durability, the PP-TENG demonstrated better results. Such outcomes originate from the fact that the dome-shaped nanostructure deforms more than the pillar-shaped nanostructure.

**Fig. 9:**
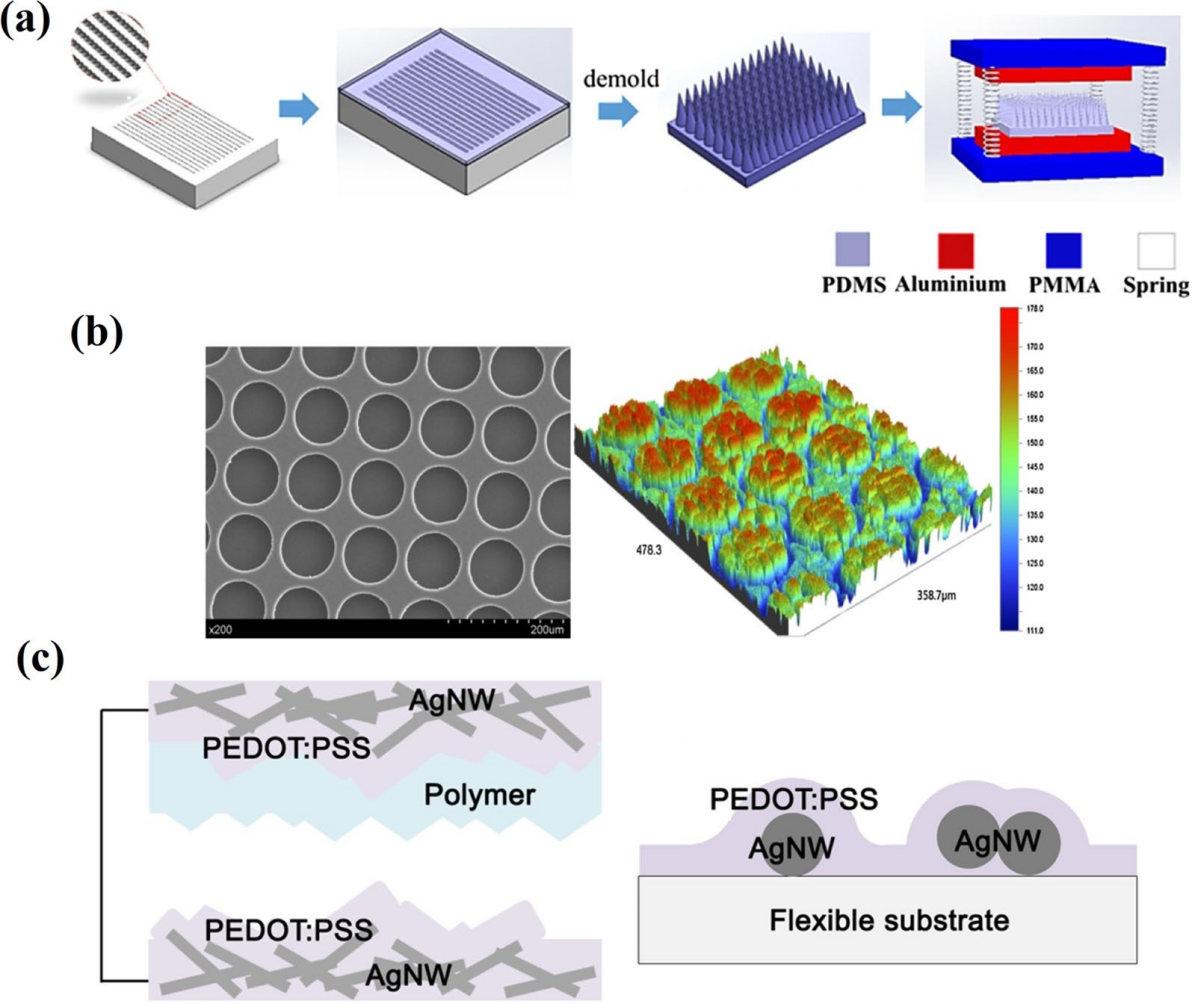
Surface micro-/nano-patterning techniques: **a** a schematic fabrication of the overlapped microneedles arrayed PDMS and the assembly of Al/PDMS MN-TENG (Reproduced with permission from Ref. [71]); **b** SEM image of the patterned PDMS before plasma treated and the topography of the pattern PDMS film treated by plasma (Reproduced with permission from Ref. [72]); **c** diagrams showing TF-TENG with embedded AgNWs in the PEDOT:PSS layer and the enlarged cross-sectional view of PEDOT:PSS/AgNW layer on a substrate (Reproduced with permission from Ref. [73])

Chung et al*.* [71] proposed a new morphology of overlapped microneedles (OL-MN) arrays in order to increase the contact surface area of Al/PDMS-TENG under low operation frequencies generated using hand tapping. The schematic fabrication of the overlapped microneedles arrayed PDMS and Al/PDMS MN-TENG assembly is illustrated in Fig. 9a. The low density (LD-MN-TENG) and high density (HD-MN-TENG) separated array types of the microneedle density-based TENGS were used as references for comparison with the overlapped microneedle density TENG (OL-MN-TENG). The OL-MN-TENG exhibits better performance compared to the LD-MN-TENG and HD-MN-TENG. OL-MN-TENG exhibited *V*_*oc*_ and *I*_*sc*_ of 123 V and 109.7 μA,

respectively. Cheng et al. [72] applied argon plasma to etch the PDMS surface to introduce microstructures on its surface without a chemical modification (Fig. 9b). The surfaces with different roughness properties were obtained by varying the parameters, such as treatment time and plasma power. The effect of plasma power on the etching efficiency of PDMS and consequent output performance was determined. Thus, *V*_*oc*_ and *I*_*sc*_ increased with the etching time when the power was 60 W. However, for higher powers, i.e., 90 and 120 W, *V*_*oc*_ and *I*_*sc*_ initially increased and then decreased with etching time. Many evenly distributed micropillars appeared on the PDMS surface at a plasma power of 90 W and treatment time of 5 min. The corresponding TENG with the plasma-treated smooth surface demonstrated 2.6 times higher output performance than the one without treatment. It should be noted that the output performance decreased to about 2/3 of the original performance after 3 months, which indicates that the argon plasma treatment was only temporarily efficient. The performance degradation was attributed to the hydrophobic recovery properties of PDMS.

Lee et al*.* [73] developed a transparent and flexible TENG (TF-TENG) based on a metallic nanowire-embedded conducting polymer as the contact electrode. A highly tribo-negative conducting polymer, i.e., poly(3,4-ethylenedioxythiophene):poly(styrene sulfonate) (PEDOT:PSS), served as the contact layer as well as the electrode material. Further, conductivity and surface roughness of PEDOT:PSS has been improved by embedding a silver NW (AgNW) layer, as shown in Fig. 9c. Polyurethane acrylate (PUA) was utilized as the counter contact material due to its strong charge-donating property, high transparency and flexibility. The surface of PUA was also micro-structured using a fabric that possesses a natural roughness through imprinting by soft lithography in order to increase the effective contact area. The corresponding TENG has the output voltage and current of about 160 V and 50 µA, respectively. TENG based on PDMS having a well-ordered nest-like morphology demonstrated a maximum output of 271 V and 7.8 μA, respectively, which are 3.7- and 2.1 times those of flat PDMS film-based TENG [65]. The enhanced performance, related to the ordered pores on PDMS, was fabricated by hydrochloric acid etching of 500 nm-sized ZnO spheres made of aggregated nanoparticles, and having a light transmittance of 81.8% and a water contact angle of 118.62°. The investigation of stability and durability of FT-TENG demonstrated a negligible change in the output current over 1000 cycles.

### Surface Chemistry Functionalization

**Fig. 10:**
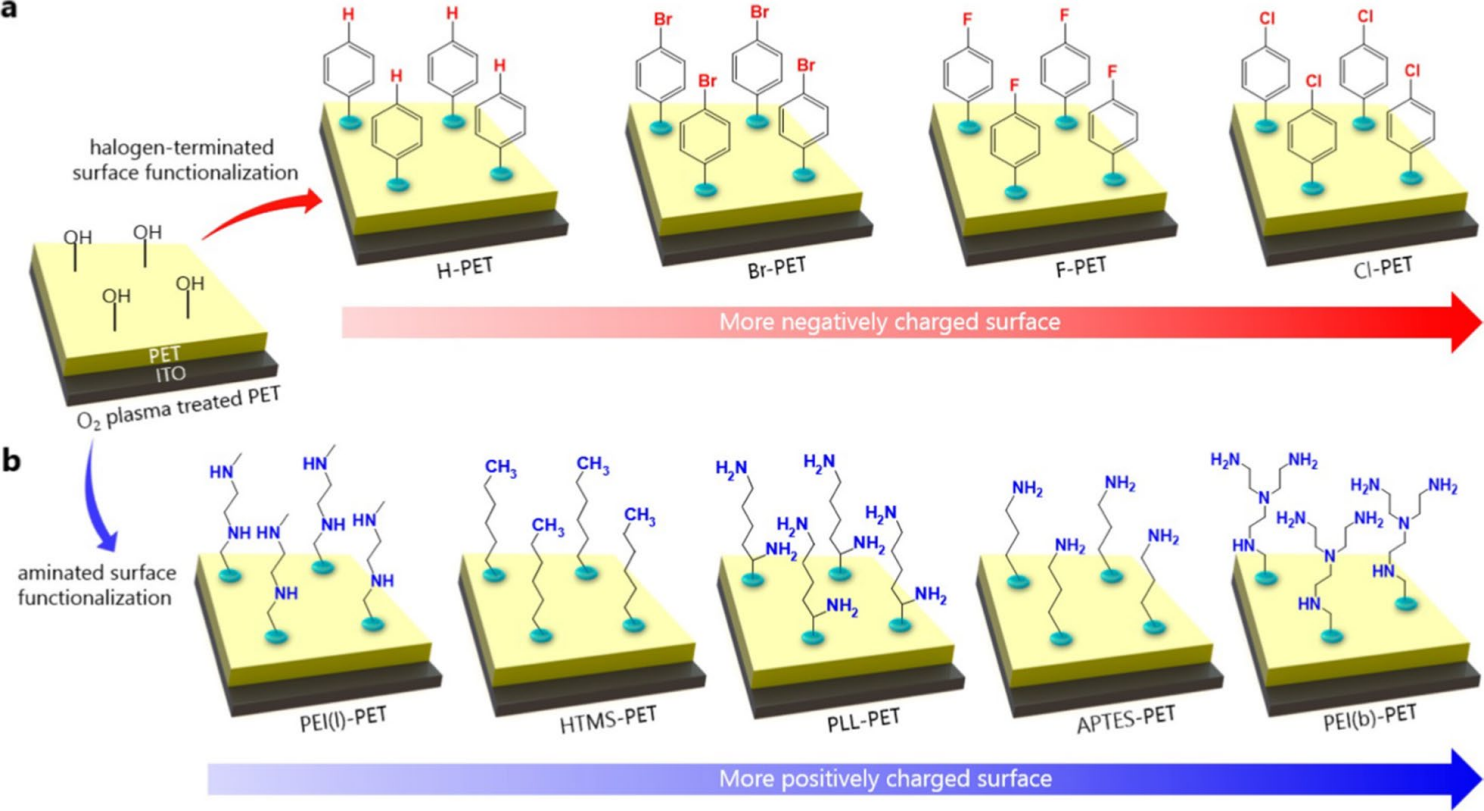
Schematic representations of surface functionalized negative and positive PETs with adopted molecules. The PET/ITO substrates were treated by O_2_ plasma to form hydroxyl (–OH) groups on the PET surface for strong hydrogen bonding with target molecules. **a** PET surfaces were functionalized with halogen (Br, F, and Cl)-terminated phenyl derivatives for negatively charged surfaces. **b** Aminated molecules, such as linear polyethyleneimine (PEI(l)), hexyltrimethoxysilane (HTMS), poly-L-lysine (PLL), 3-aminopropyltrimethoxysilane (APTES), and branched polyethylenimine (PEI(b)), were functionalized on the O_2_ plasma treated PET surfaces (Reproduced with permission from Ref. [77])

A significant difference in the triboelectric polarity between the contact layers can be achieved by chemical modification. The practical and straightforward approach to modify the surface potential is to use a chemical surface functionalization since the contact electrification's driving force is closely related to the chemical potential difference between the two surfaces [74]. Because the chemical potential of organic polymeric material is directly related to its functional groups, the surface potential can be favorably regulated by introducing appropriate functional groups exposed on the surface without changing the bulk material and its main properties [75]. It is known that most electron-attracting polymers contain groups with strong electron affinity like fluoro-groups (-F), while amine (-NH_2_) ones have the lowest affinity. Thus, it is expected that introducing extra -F atoms onto the polymer surface by chemical modifications is a way to increase charge density [76]. Shin et al*.* [77] demonstrated a simple approach to change the triboelectric property of a polymeric surface via atomic-level chemical functionalization using a series of halogens and amines, which results in a broad spectrum of triboelectric series over a single material such as PET. The authors varied the chemical structure of a surface functional group while keeping the other components constant; this allowed them to relate these components to the performance of the corresponding TENG and investigate the mechanism of a triboelectric process on the chemically functionalized surfaces. Arylsilanes terminated with halogens were introduced to induce triboelectrically negative properties on the PET surface while several aminated molecules were applied for the tribo-positive functionalization (Fig. 10). Remarkably, the surfaces functionalized with triethoxy(4-chlorophenethyl)silane and branched polyethyleneimine were revealed as the most triboelectrically negative and positive ones, respectively. This work demonstrated the possibility of introducing various functional groups onto materials through a straightforward surface chemical functionalization and creating TENG materials with finely tunable triboelectric properties.

**Fig. 11:**
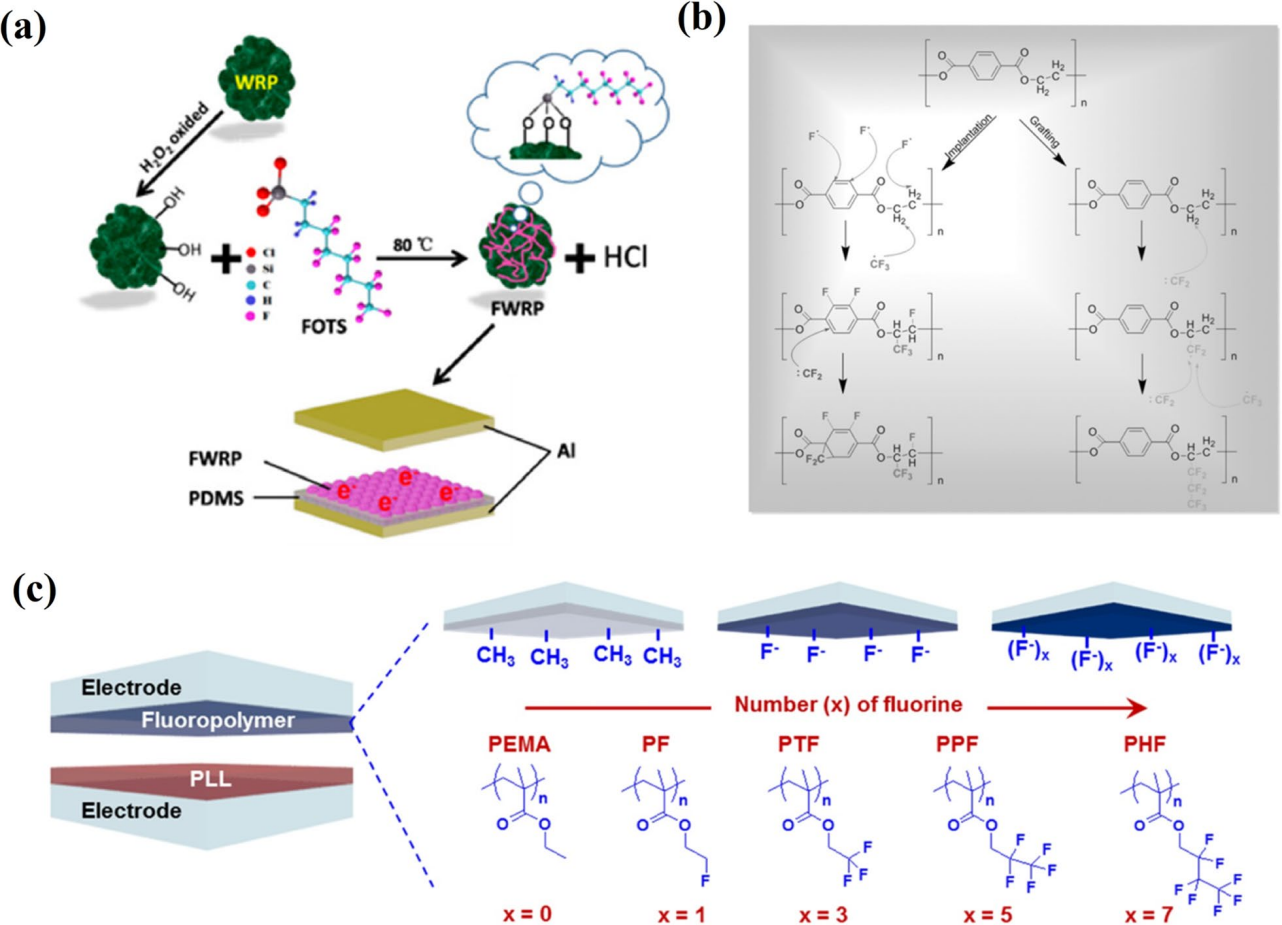
Chemical functionalization of TENG surface layer: **a** schematic representation of surface fluorinated modification of WRP by FOTS (Reproduced with permission from Ref. [78]); **b** schematics illustrations of the reactions of fluorination on a PET surface (Reproduced with permission from Ref. [79]); c a schematic of TENG based on the fluorinated polymers with different kinds of fluorine units (Reproduced with permission from Ref. [80])

The contact-separation mode TENG, with a high output performance where surface fluorinated wasted rubber powder (WRP) was used as source material, has been reported (Fig. 11a) [78]. The surface modification with (1H,1H,2H,2H-perfluorooctyl)silane (FOTS) enhanced the output performance of WRP-based TENG, showing a maximum of which *V*_*oc*_ = 265 V and a *J*_*sc*_ = 75 mA/m^2^ enabled it to power 100 commercial LEDs directly. Moreover, the fabrication of the abovementioned TENG, which is simple in preparation, stable and not expensive, shows a possibility for the extension of waste utilization like wasted rubber. An inductive-coupled plasma etched by a mixture of the carbon tetrafluoride (CF_4_) and oxygen (O_2_) gases of the surface of a PET film-based TENG increased the triboelectric charge density. The prepared TENG demonstrated a large improvement in *V*_*oc*_, *I*_*sc*_, and induced charge quantity by 300%. An enhancement in the triboelectric charge density is attributed to both chemical modification by fluorination and physical modification by roughened surface morphology (Fig. 11b) [79].

TENG based on the modified PET film as the contact material demonstrated superior electric output compared to the one without a modification. Kim et al. in [80] studied the relationship between the molecular structures of fluorinated polymer films and the resulting triboelectric output performance together with relative dielectric constants and triboelectric polarity. Fluorinated polymers with a controlled fluorine unit and molecular weight (*M*_*w*_) were synthesized by a reversible addition-fragmentation chain-transfer (RAFT) polymerization. Figure 11c illustrates a triboelectric device using fluorinated polymers with different fluorine units and poly (L-lysine) (PLL) as negative and positive triboelectric materials, respectively. Poly(ethyl methacrylate) (PEMA) was used as a reference polymer, with the same chemical backbone as fluorinated polymers but without fluorine units. The investigations revealed a direct relationship between the dielectric constant and fluorine units in fluorinated polymers. Thus, the relationships of the molecular chain structures of the fluorinated polymers, *M*_*w*_, and conditions (such as spin rate and annealing temperature with the dielectric constants of dielectric layers and the triboelectric polarity) closely related to the triboelectric output performance were observed. For instance, the dielectric constant increased as the number of fluorine units increased in the polymers from zero to three. Still, a slight decrease was observed when the number of fluorine units became more than three, which matched the results of the triboelectric output currents. A poly(2,2,2-trifluoroethyl methacrylate) polymer with three fluorine units and *M*_*w*_ of about 20 kg/mol demonstrated better triboelectric output performance than the other studied fluorinated polymers.

**Fig. 12:**
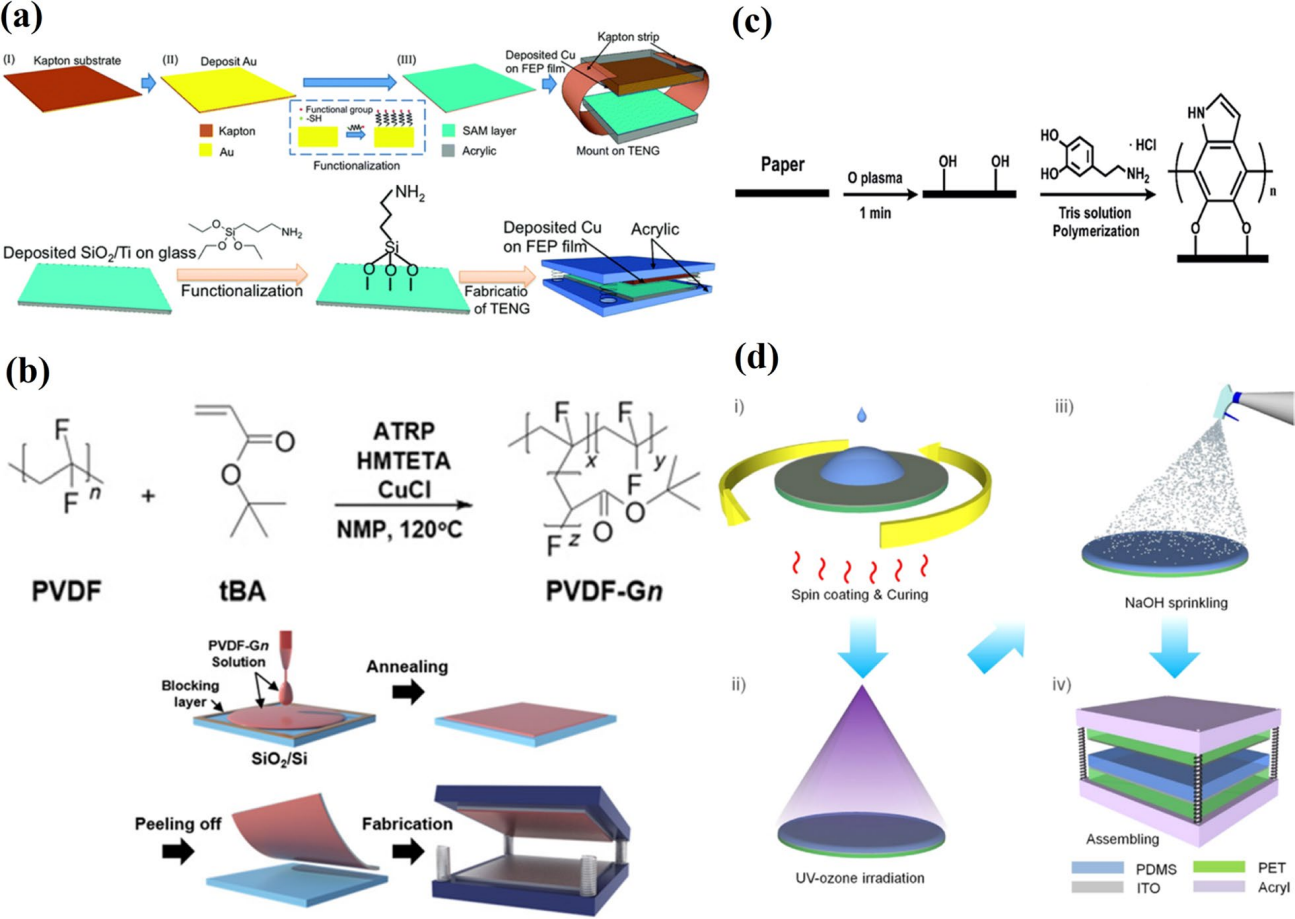
Chemical functionalization of TENG surface layer. **a** Fabrication process of TENGs from the thiol-SAM functionalized Au films (i) and from SAM surface functionalized SiO2 (ii) (Reproduced with permission from Ref. [82]). **b** Synthesis of PVDF-Gn graft copolymers and schematic diagram of the fabrication process for the PVDF-Gn-based TENG (Reproduced with permission from Ref. [83]). **c** Schematic diagram of polydopamine modification of paper (Reproduced with permission from Ref. [84]). **d** Schematic illustration of surface treatment of polydimethylsiloxane (PDMS) polymer film and a TENG device (Reproduced with permission from Ref. [85])

The self-assembled (SAM) monolayer method is one of the important ways of surface morphology engineering, in which ordered molecular assemblies are formed by the chemical adsorption of an active surfactant on a solid surface [81]. Wang et al. [82] reported the approach to increase the triboelectric charge density by a chemical surface functionalization through the two types of self-assembled monolayers, i.e., thiols and silanes, to modify the surfaces of conductive (Au) and insulating (SiO_2_) materials, respectively. The four different functional groups such as hydroxyl (–OH), ester (–COOCH_3_), amine (–NH_2_) and chloro (–Cl) were inserted onto the Au surface during the thiol-based (Fig. 12a(i)) and onto the SiO_2_ surface (Fig. 12a(ii)) during the silane-based SAM functionalization processes. FEP film was chosen as a tribo-negative material and the surface potential was investigated using scanning Kelvin probe microscopy in order to determine the influence of head groups. The studies revealed that amine (-NH_2_) is the most tribo-positive functional group and produced the most significant improvement in the generated charge density. The corresponding TENG’s output performance was improved accordingly. An effective dielectric material for the TENG fabrication with high performance is crucial because the output power is highly dependent on the density of the charges transferred. Lee et al. [83] reported an effective approach to improve the output power of TENG with the successful synthesis of poly(tert-butylacrylate)(PtBA)-grafted PVDF copolymers via a dielectric constant control through an atom-transfer radical polymerization (ATRP) technique (Fig. 12b). The method of grafting one polymer onto another polymer backbone allows for the combination of the favorable properties of each parental polymer. An increase in dielectric constant values of the copolymer by approximately twice was attributed to *α*-phases with enhanced dipole moments due to the π-bonding and polar characteristics of the ester functional groups in the PtBA. These characteristics lead to increased density of the charges accumulating on the copolymer during physical contact. The dielectric constant values went up from 8.6 to 16.5 for the frequency varying from 10^2^ to 10^5^ Hz, and as the grafting ratio increased to 18% due to the increased net dipole moment of the materials. Kelvin probe force microscopy measurements supported this. The graft copolymer-based TENG demonstrated a *V*_*oc*_ and *J*_*sc*_ of 64.4 V and 18.9 mA/cm^2^, respectively. This is a twice better performance compared to the pristine PVDF-based TENG.

Moreover, a 20-fold improvement in the output power was achieved by poling the film to align the dipole direction, which enhanced the charge-accepting characteristics by increasing the work function of the copolymer. An improvement in a paper-based TENGs output performance was reported where polydopamine molecules (a high polar agent) were introduced to increase the surface polarity of the friction layer by a simple self-polymerization reaction at room temperature (Fig. 12c) [84]. Here, TENG was fabricated from gum wrappers (a friction layer) coated with an Al foil as the conduction layer. After the chemical modification, *I*_*sc*_ and *V*_*oc*_ were found to increase by as much as 3.5 times, with maximum values of 30 μA and 1000 V reported, respectively. The enhancement of the output performance of PP-TENG was attributed to the aspherical nanostructures formed after a modification with polydopamine. A simple approach to improve a triboelectric surface charge of the PDMS surface by irradiation with ultraviolet-ozone (UVO) followed by a sprinkling of 1 M NaOH solution was demonstrated by Yun et al. [85]. After sprinkling the PDMS, the surface with 1 M NaOH solution TENG generated 10.4 V and a current of 179 nA. The fresh PDMS-based TENGs generated a *V*_*oc*_ of 3.8 V and a *I*_*sc*_ of 65 nA. It is clear that the irradiation of the PDMS by UVO prior to sprinkling with NaOH solution resulted in better triboelectric power output as the voltage and current increased to 49.3 V and 1.16 μA, respectively. This is almost a 15-fold enhancement with respect to the fresh PDMS. Figure 12d illustrates the surface treatment procedure used on the PDMS films. This improvement in TENG’s output was related to the chemical modification of non-polar Si-CH_3_ bonds to polar Si–O bonds in PDMS due to the treatment with UVO and NaOH solution.

**Fig. 13:**
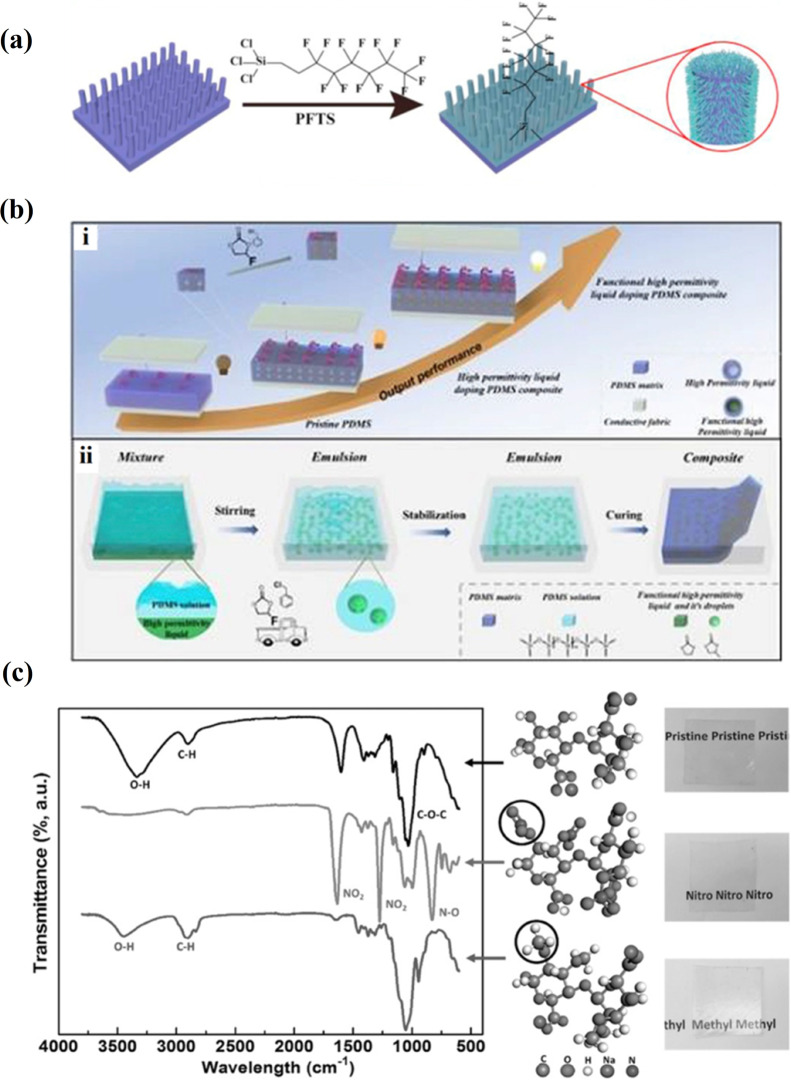
Chemical functionalization of TENG surface layer. **a** Schematic depiction of the surface modification with fluorinated compounds of the PP nanowires prepared by hot processing technique (Reproduced with permission from Ref. [86]). **b** i—Scheme of functional high permittivity liquid doping PDMS composite; ii—Fabrication of high permittivity liquid doping PDMS composite and fluorine functionalization (Reproduced with permission from Ref. [87]). **c** FTIR spectrum and molecular structure of pristine CNF (top row), nitro-CNF (middle row), and methyl-CNF (bottom row). Photos of the transparent films fabricated from these three cellulosic materials (Reproduced with permission from Ref. [63])

A chemical functionalization with a 1H,1H,2H,2H-perfluorooctyltrichlorosilane modifier of the polypropylene (PP) nanowire array-based TENG increased *V*_*oc*_ to 1900 V and *J*_*sc*_ to 19 mA/m^2^ (Fig. 13a) [86]. Compared with the smooth PP film-based TENG, the *J*_*sc*_ of the chemically modified TENG increased by more than 100 times and was found to light up 372 commercial LEDs. The fluoroethylene carbonate (FEC) additive for fluorine functionalization and the benzyl chloride (BC) additive for chloride functionalization were applied during the preparation of composite triboelectric materials where a high-permittivity liquid, serving as functional molecules micro-carrier, was dispersed in the PDMS matrix by emulsion mixing (Fig. 13b) [87]. Given that a liquid is highly soluble, the functionalized PDMS composite was easy to fabricate from FEC or BC functional additive dissolved liquid, avoiding the functional additive's necessity of chemical reactivity. This indicated great extensibility in functionalized triboelectric materials. Compared to the pristine PDMS, 5.7-fold, 5.4-fold and 44.8-fold enhancements in the output voltage, current and power density, respectively, were demonstrated by the fluorine functionalized composite, i.e., PDMS-FHD45. This work demonstrated that a functional additive liquid doping composite is a promising method for functionalizing triboelectric materials with great extensibility. The cellulosic materials were used to fabricate a high‐performance triboelectric pair for TENG. The raw cellulosic material was used to fabricate both positive and negative tribo-materials for TENG. CNFs were chemically functionalized by attaching nitro- and methyl groups to their molecules in order to change the tribopolarities of CNF (Fig. 13c) [63]. The corresponding tribo-pair demonstrated high performance. The nitro-CNF possesses a negative surface charge density of 85.8 µC/m^2^, while the methyl-CNF possesses a positive surface charge density of 62.5 µC/m^2^. TENG fabricated from nitro-CNF paired with methyl-CNF showed an average voltage output of 8 V and a current output of 9 µA, respectively.

### Charge Injection, Trapping and Enhancing Dielectric Constant

**Fig. 14:**
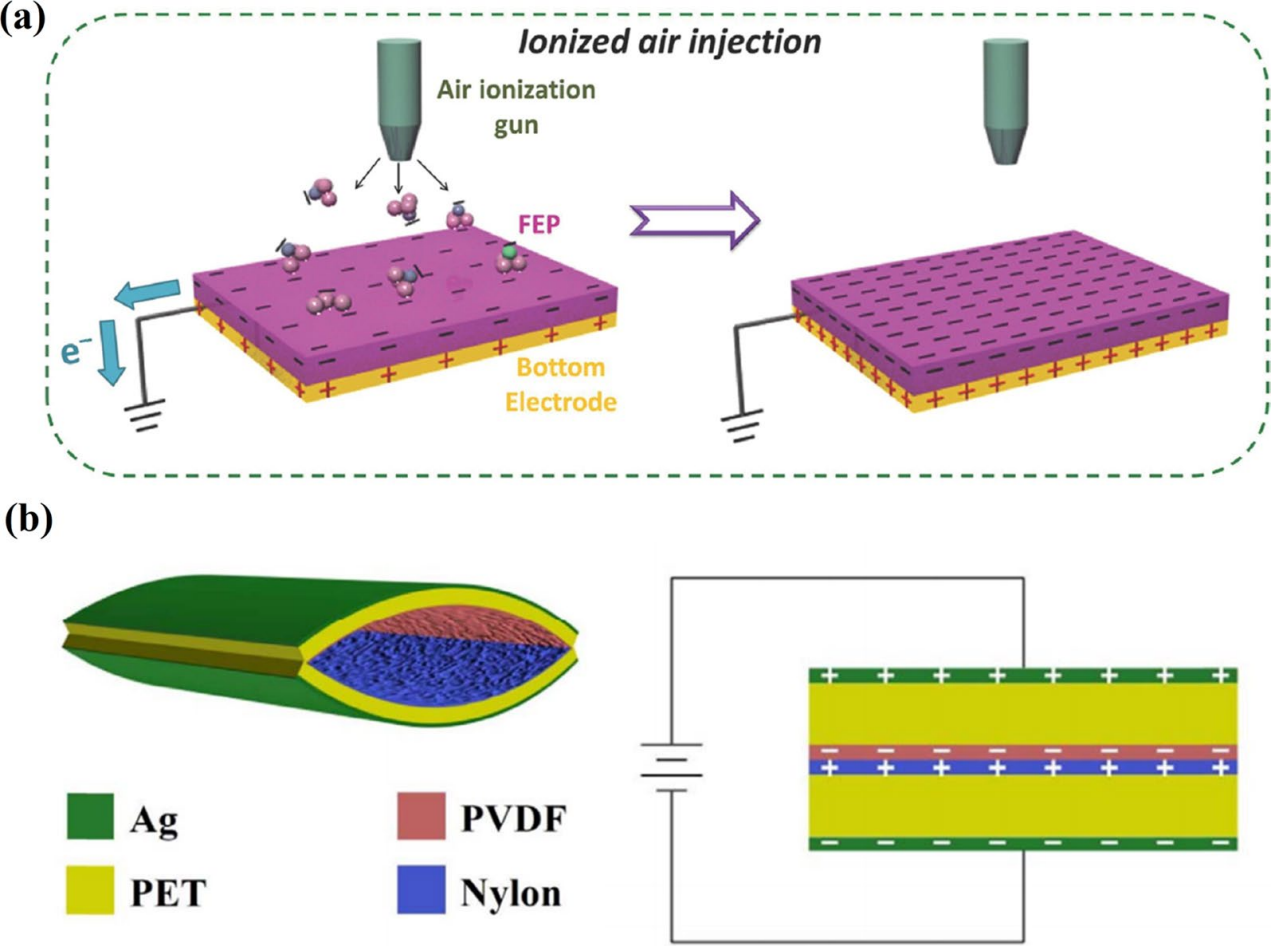
Charge injection techniques: **a** a process of the ion injection on the FEP film (Reproduced with permission from Ref. [89]); **b** a structure of the high-performance TENG and schematic image of the equipment used for charge injection (Reproduced with permission from Ref. [90])

Charge injection is an approach to improve the output performance of triboelectric energy harvesters. An enormous amount of the triboelectric charges transferred on the surface of the contact materials via the charge injection or trapping can lead to a strong driving force for the high-output voltage and current [88]. A simple and effective method to increase surface charge density is the direct injection of single-polarity charged particles and ions onto the contact surface. Wang et al*.* [89] proposed a process of injecting single-polarity charged particles/ions onto the surface of an electret to improve the output performance of TENGs. An air-ionization gun was used to inject charges onto the material surfaces, and the polarities of the ions were triggered by the discharge of air inside the gun (Fig. 14a). The negative ions like CO_3_^−^, NO_3_^−^, NO_2_^−^, O_3_^−^ and O_2_^−^ were injected onto the surface of fluorinated ethylene propylene, which improved the output of TENG by 25 times. Also, for the first time, by repeating the ion-injection process multiple times, the maximum surface charge density for TENG due to the limitation of the air breakdown was confirmed. A cheap and straightforward method of introducing nanostructures on contact surfaces was conducted by direct charge injection into the space between the surface of the friction layers [90]. Nanostructures on the surface of nylon and PVDF film, chosen as tribo-materials, were formed during the spin-coated process on the PET film as the solvents evaporated rapidly. The dielectric breakdown phenomenon was applied as a charge injection method, where an air gap breakdown occurred between the nylon and PVDF film due to the dielectric constant of the air gap being smaller than nylon and PVDF (Fig. 14b).

Afterward, the ionized positive and negative charges transferred into the friction layers due to the direction of an external electric field. The injected charges work along with the triboelectric charges to improve the performance of TENG. Introducing nanostructures improved the charge density by 48%, while the charging injection improved by 53%. *V*_*oc*_ and *J*_*sc*_ reached 1008 V and 32.1 mA/m^2^, respectively.

**Fig. 15:**
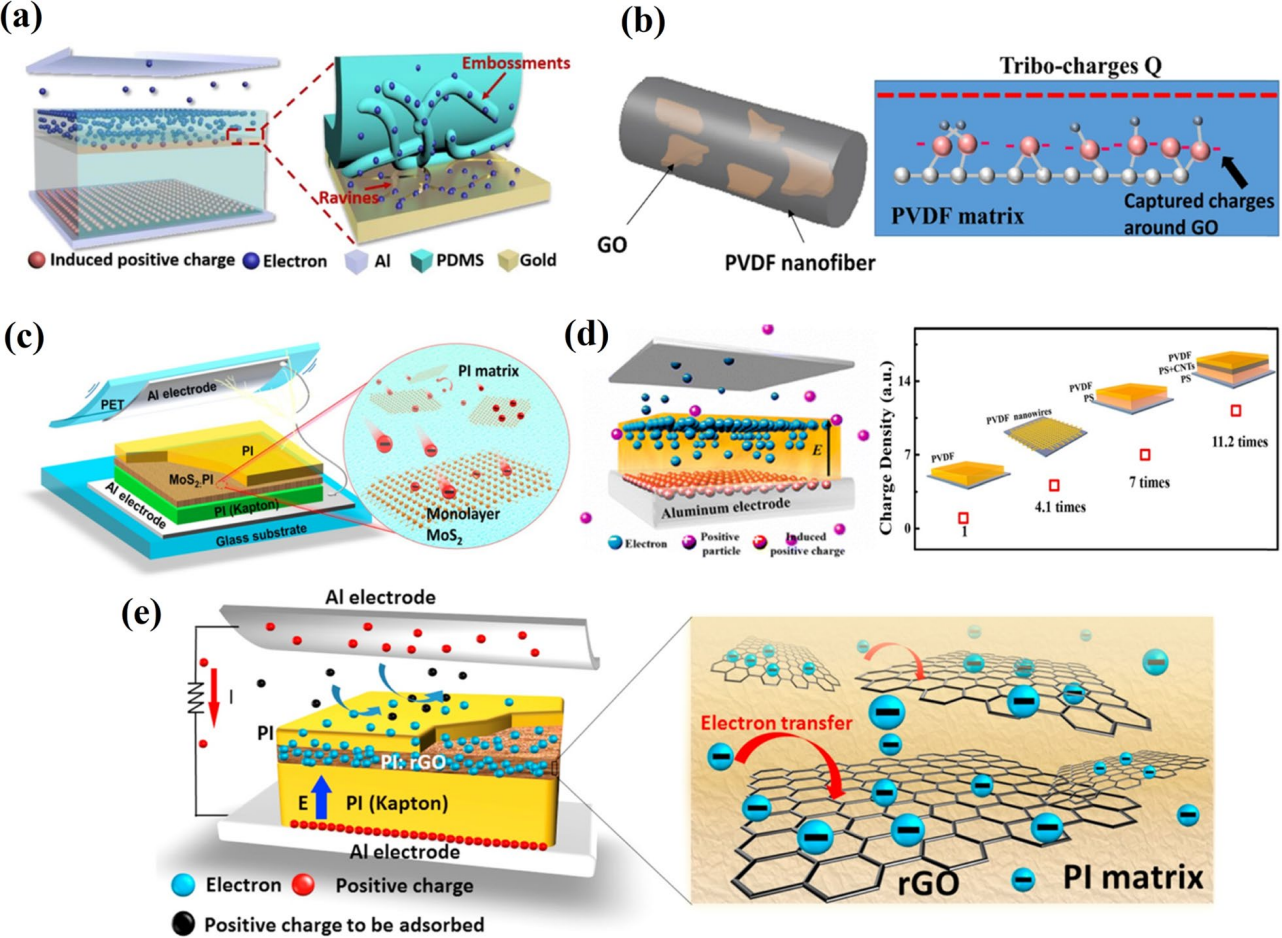
Different approaches applied for charge trapping. **a**) Illustrations of electrons’ drift in G-TENG and the schematic diagram of electrons’ escape from PDMS to the gold (Reproduced with permission from Ref. [91]). **b** Distribution of GO on a nanofiber and stored charge on the surface of a GO sheet (Reproduced with permission from Ref. [94]). **c** Illustration of the vertical contact-separation mode TENG with a MoS_2_-monolayer film and a schematic diagram of the electron transfer from the PI layer to the MoS_2_ monolayer (Reproduced with permission from Ref. [97]). **d** Schematic of the transport process of triboelectric electrons in the negative friction layer of a TENG and improvement effects of different composite friction layer structure (Reproduced with permission from Ref. [98]). **e** Illustration of the vertical contact-separation mode TENG with a PI:rGO film and a schematic diagram of electron transfer from the PI layer to the rGO sheets (Reproduced with permission from Ref. [99])

Trapping the charges inside triboelectric materials increases the output performance of TENG due to the suppression of the loss of triboelectric electrons by the trapping sites [91]. It is essential to block a charge combination that originates when the triboelectric surface potential at one surface induces the opposite electrostatic potential at the interface with the counter electrode. An electric field can cause the surface charges to be combined with the induced opposite charges leading to the interference of this charge combination with a surface charge accumulation, so bringing a drastic decrease in the triboelectric potential [92]. Figure 15a shows plasma treatment used to trap triboelectric charges through embedding ravines and gullies and crisscrossed gold layers in the near-surface of the tribolayer [91]. Experimental results demonstrated that the transfer charge density of prepared TENG was nearly four times the TENG value without passageways and traps. Prada et al*.* [93] modified the surface in PTFE TENG by a powerful O_2_ and Ar bipolar plasma etching without CF_4_. The surface-modified TENG demonstrated a power density of 9.9 W/m^2^, which was about thirty times higher than unmodified PTFE TENG. The enhanced power output was attributed to increased surface area and charge trapping sites developed during sequential O_2_/Ar plasma etching.

Several reports utilized the GO sheets as trapping sites. A book-shaped TENG fabricated from electrospun PVDF and poly(3-hydroxybutyrate-co-3-hydroxyvalerate) (PHBV) nanofibers with dispersed GO in the PVDF nanofibers acting as charge trapping sites was reported (Fig. 15b) [94]. The GO sheets improved the charge storage ability of the PVDF nanofibers and increased the output performance of the TENG. The maximum output peak-to-peak voltage and the current were 340 V and 78 μA, respectively. Layered MoS_2_ and its composites have properties like a large specific surface area and appropriate energy level, along with the quantum confinement effect, suggesting these as promising charge-trapping material [95, 96]. It was reported that liquid-phase exfoliated 2D monolayer MoS_2_ can act as triboelectric electron acceptors in TENGs and significantly enhance their output performance [97]. That significant improvement in the electricity output of TENGs was attributed to the efficient electron capture in the monolayer MoS_2_, which can suppress recombination between triboelectric electrons and positive charges (Fig. 15c). The peak power density of TENGs with a 2D monolayer MoS_2_ was 25.7 W/m^2^, which was 120 times larger than that of TENG without monolayer MoS_2_. Cui et al*.* [98] studied the storage mechanism of triboelectric charge in the friction layer and discussed the function of carrier mobility and concentration in the charge storing process. A composite structure was embedded in the friction layer to adjust the depth distribution of the triboelectric charges and improve the output performance of TENGs. Thus, a simple TENG was fabricated using the PVDF as a negative friction layer with an integrated composite structure consisting of PS and carbon nanotubes (CNTs) (Fig. 15d). This composite friction layer improved the triboelectric charge density by a factor of 11.2 compared to the pure PVDF friction layer. Wu et al. [99] proposed a promising approach for improving the output performance by introducing electron traps into the PI flat film friction layer (Fig. 15e). Reduced GO (rGO), a graphene derivative consisting of a hexagonal carbon network with sp^2−^ and sp^3−^ hybridized carbon atoms was used to trap electrons on the friction layers. The rGO-based TENG reached a maximum power density of 6.3 W/m^2^, which was 30 times higher than that of TENG without rGO. It also drove a more considerable amount of charge through the external load per cycle favorable for charging energy-storage devices. A significant effect of the thin parylene-C film in improving the output performance of TENG by a factor of 10 when introduced on bare PTFE film was reported by Ravichandran et al. [100]. The parylene-C film was deposited to improve the charge accumulation by receiving more charges, thus enhancing the surface charge density by up to 70.2 µC/m^2^, which is 140 times higher than that of the initial results.

Enhancing the dielectric properties of tribo-materials is another effective way of increasing surface charge density. Suphasorn et al*.* [101] incorporated Ag NPs into natural rubber (NR) to increase the dielectric constant. As a result, the dielectric constant of the Ag-added NR films increased up to 40%. In this work, the NR@AgNPs TENG with AgNPs was synthesized by a chemical reduction method of AgNO_3_ using NaBH_4_ as a reducing agent and 1.5 mM cetyltrimethylammonium bromide (CTAB) as a surfactant and demonstrated the highest power density of 262.4 mW/m^2^.

Composite film for TENG fabrication with optimized dielectric constant leading to improved charge density and internal capacitance was developed using thermoplastic polyurethane (TPU) matrix with polyethylene glycol (PEG) additives and PTFE NP inclusions [102]. The composite film-based TENG output power demonstrated about 17 times enhancement reaching 16.8 mW at an external resistance of 200 kΩ. Moreover, a 90% decrease in matching impedance was observed.

PS microspheres increased the contact area of the friction layer (PVDF-TrFE) and improved permittivity due to the Maxwell–Wagner-Sillars polarization originating from the accumulation of charge carriers at the heterogeneous system interfaces due to different conductivity between fillers and matrix [103]. Moreover, the positively charged amino groups of the hexadecyl-trimethyl-ammonium-bromide were grafted on PS microspheres. The modified TENGs demonstrated better output performance than TENG from pristine PVDF-TrFE. A maximum output power density close to 8 W/m^2^ under 5 Hz and 50 N periodic force was achieved.

Shao et al*.* [104] introduced BaTiO_3_ particles into TENG based on bacteria cellulose film. The simultaneous improvement in the dielectric constant and surface area of the film resulted in 150 and 210% enhancement in open voltage and short circuit current, respectively. The TENG with 13.5% BaTiO_3_ embedded showed *V*_*oc*_ about 181 V and *I*_*sc*_ equal to 21 µA, a peak power density of 4.8 W/m^2^ was achieved when connecting with the resistance in series.

### Physical Hybridization with Other Energy Harvesting Technologies

Strategies for improving the output power of TENG have never been restricted to only the development of new materials, increasing the surface roughness and chemical modification. An efficient approach to harvest simultaneously multiple types of energy is the amalgamation of different types of energy harvesting technologies that enhance the output performance of a hybrid device. Thus, for example, TENG integration with an EMG allows generating electricity at low mechanical frequencies, where EMG alone is not efficient. On the other hand, TENG fabricated from pyroelectric materials can generate electricity from the ambient thermal energy or even from a temperature increment caused by the friction of triboelectric layers. The advantage of PENG is the ability to provide a high conversion efficiency and stable output power under weak and low-frequency mechanical stimuli. The combination of both the piezoelectric and triboelectric effect is possible without any complex device structure; the only requirement is that one of the materials chosen for TENG should also be a piezoelectric material.

**Fig. 16:**
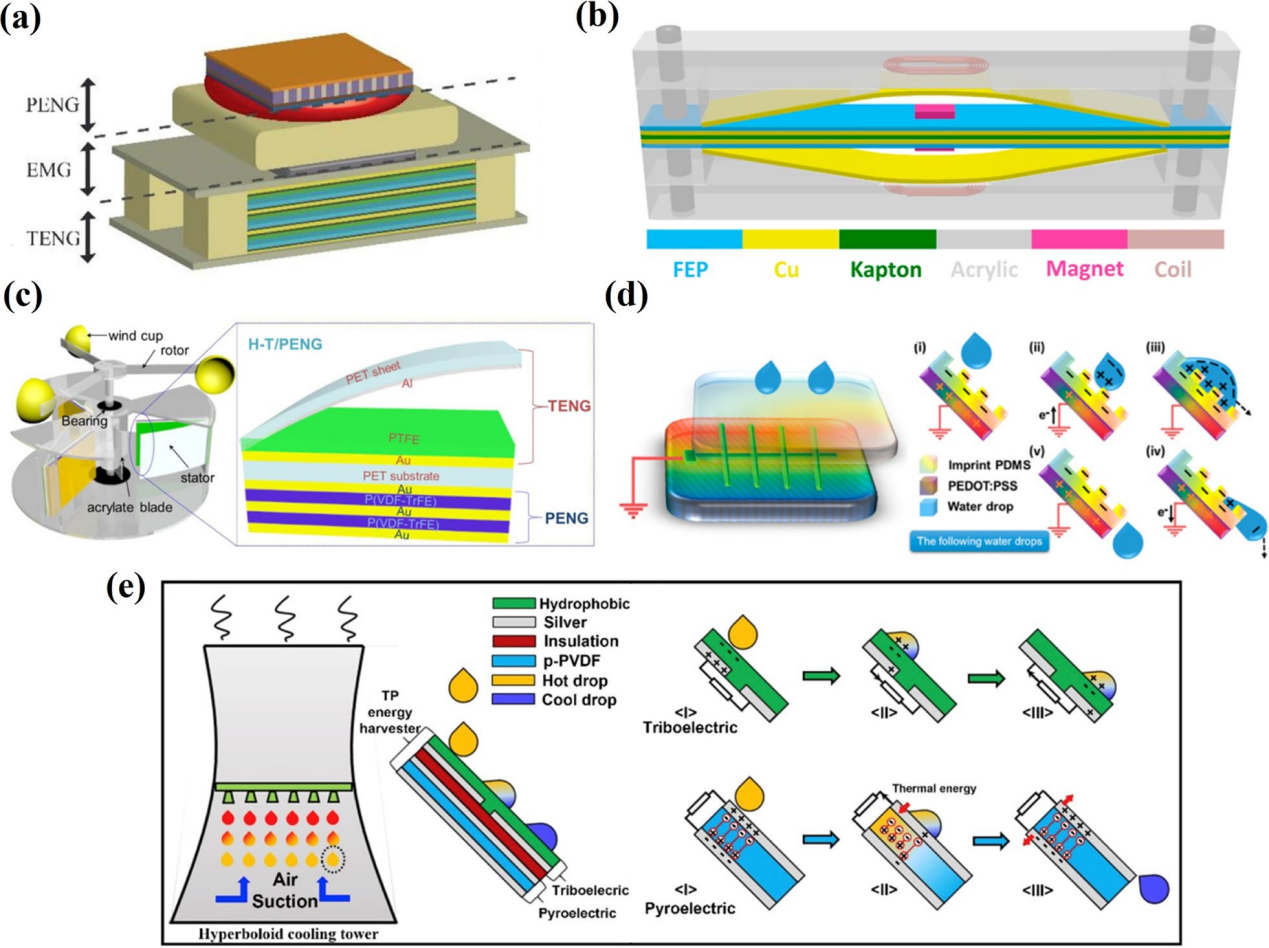
Hybrid devices based on TENG. **a** Schematic illustration of the hybrid generator (Reproduced with permission from Ref. [105]). **b** Schematic diagram of the fabricated hybridized EM-TENG (Reproduced with permission from Ref. [106]). **c** Schematic illustration of the H-P/TENGs mounted in the custom frame and the enlarged structure of single H-P/TENG (Reproduced with permission from Ref. [107]). **d** Schematic illustration of a typical TENG structure and working mechanism of TENG (Reproduced with permission from Ref. [108]). **e** Schematic diagram of the TPENG and working principle of the triboelectric, and pyroelectric generators during interaction with a hot water droplet (Reproduced with permission from Ref. [109])

Rodrigues et al*.* [105] demonstrated a hybridized energy harvesting device based on TENG, EMG and PENG embedded to a shoe sole (Fig. 16a). The TENG structures such as parallel, arched and zigzag triboelectric plates working at contact-separation mode were studied. Among these structures, the parallel-plate-based TENG exhibited the highest performance. Further, the distance between the triboelectric layers and the number of tribo-pair for corresponding TENG was also optimized, resulting in a significant improvement in the output performance, enabling the charge of different capacitors. Finally, an effective conjugation of triboelectrification, electromagnetic induction and piezoelectricity resulted in hybridized NG with improved energy generation properties that increased by 20% the charging capacity of the TENG system alone. Hybridized EMG-TENG depicted in Fig. 16b simultaneously scavenged the wind energy where TENG and EMG delivered the largest output powers of about 1.7 mW under a loading resistance of 10 MΩ, and, 2.5 mW under a loading resistance of 1 kΩ, respectively [106]. A power management circuit (PMC) was designed for efficient energy storage in the capacitor consisting of two bridge rectifiers, three capacitors, and a coupled inductor, which was necessary because of the different impedances of TENG and EMG. This resulted in an improved energy storage efficiency of up to 112% compared with that when applying a traditional rectifier. This exhibited the potential applications of hybridized NGs in self-powered sensor systems. A hybrid piezo/triboelectric NG (H-P/TENG) was reported by Zhao et al*.* [107], where bimorph-based PENG was integrated into one-end-fixed TENG. This device harvested the rotary mechanical energy from H-P/TENGs fixed on three upright posts of a customized frame exposed to bending strains from a coaxial tri-blade structure (Fig. 16c). H-P/TENG, due to the synergistic effect of triboelectrification and piezo polarization, had the identical phase and matched impedance at the same magnitude and demonstrated excellent output characteristics. *V*_*oc*_ and *I*_*sc*_ were equal to 210 V and 395 μA, respectively, at a 100 rpm rotation speed. Moreover, the output performance of H-P/TENG remained stable with a rotation speed up to 250 rpm due to the constant periodic deformation degree of H-P/TENG. A specialized energy managing circuit developed for the device allowed it to supply a stable constant output voltage (3.6 V) to power different electronic devices. Additionally, a practical application of H-P/TENG was demonstrated by harvesting energy from wind at 14 m/s providing high output voltage (150 V) and current (150 μA) for driving 50 LEDs. A hybrid of a solar cell and TENG for electricity generation from sunlight and raindrops was proposed to overcome the reduced performance of solar cells on rainy days [108]. Here, single-electrode-mode water-drop TENG was integrated with a heterojunction silicon (*Si*) solar cell through a mutual electrode of (PEDOT:PSS) film (Fig. 16d). Imprinted PEDOT:PSS in a solar cell was applied to decrease the light reflection in order to improve the *I*_*sc.*_ While in TENG, a PEDOT:PSS layer acted as an electrode combined with imprinted PDMS as a triboelectric material. The advantage of this device is the combination of the high current level of a solar cell and the high voltage of a TENG device to generate efficient energy from the environment during different weather conditions. A free-standing TENG was integrated with triboelectric PENG (TPENG) for harvesting energy from low-grade waste energy appearing in the form of thermal fluids (Fig. 16e) [109]. Apart from the thermal energy, thermal fluids also possess large kinetic energy, and the suggested device harvested energy from both thermal and kinetic energy of the thermal fluids. The hybrid device demonstrated an output power density of 2.6 μW/cm^2^ when 65 °C discrete water droplets were dripped onto it. The device was able to light up 28 commercial LEDs. Zhang et al*.* [110] fabricated a single-structure-based multi-functional coupled NG based on the piezo-tribo-pyro-photoelectric effects. The hybrid NG using the same output electrodes delivered a complementary power source with a peak current of about 5 µA, a peak voltage of 80 V and a platform voltage of 50 V, respectively. Wang et al*.* [111] demonstrated a flexible hybrid TPENG fabricated from poly(vinylidene fluoride-co-trifluoroethylene) (P(VDF-TrFE)) serving as a top functional piezoelectric layer and multi-wall carbon nanotubes (MWCNTs) dispersed into PDMS utilized as a bottom friction layer. The triboelectric and piezoelectric output peak voltages of TPENG in the open-circuit were 25 V and 2.5 V under a pressure of 5 N, respectively. Another hybrid nanocomposite-based TPENG harvester was reported by Karumuthil et al. [112]. The nanocomposite was a PDMS resin with *ZnO* nanorods, exfoliated GO and MWCNTs dispersed onto it. The device, with 5 cm × 7 cm × 1.5 mm dimensions with a thickness of PDMS nanocomposite of 1.25 mm, was applied to generate electricity from finger tapping, press-release operation and human footsteps, demonstrating an output voltage of 36 V, 40 V and 50 V, respectively, with the subsequent rectification of the obtained alternating voltages and their storage in a commercial capacitor.

## Applications

TENG has been used for two major applications: energy harvesters and self-powered sensors [12].

### Harvesting Different Kinds of Mechanical Energy

**Fig. 17:**
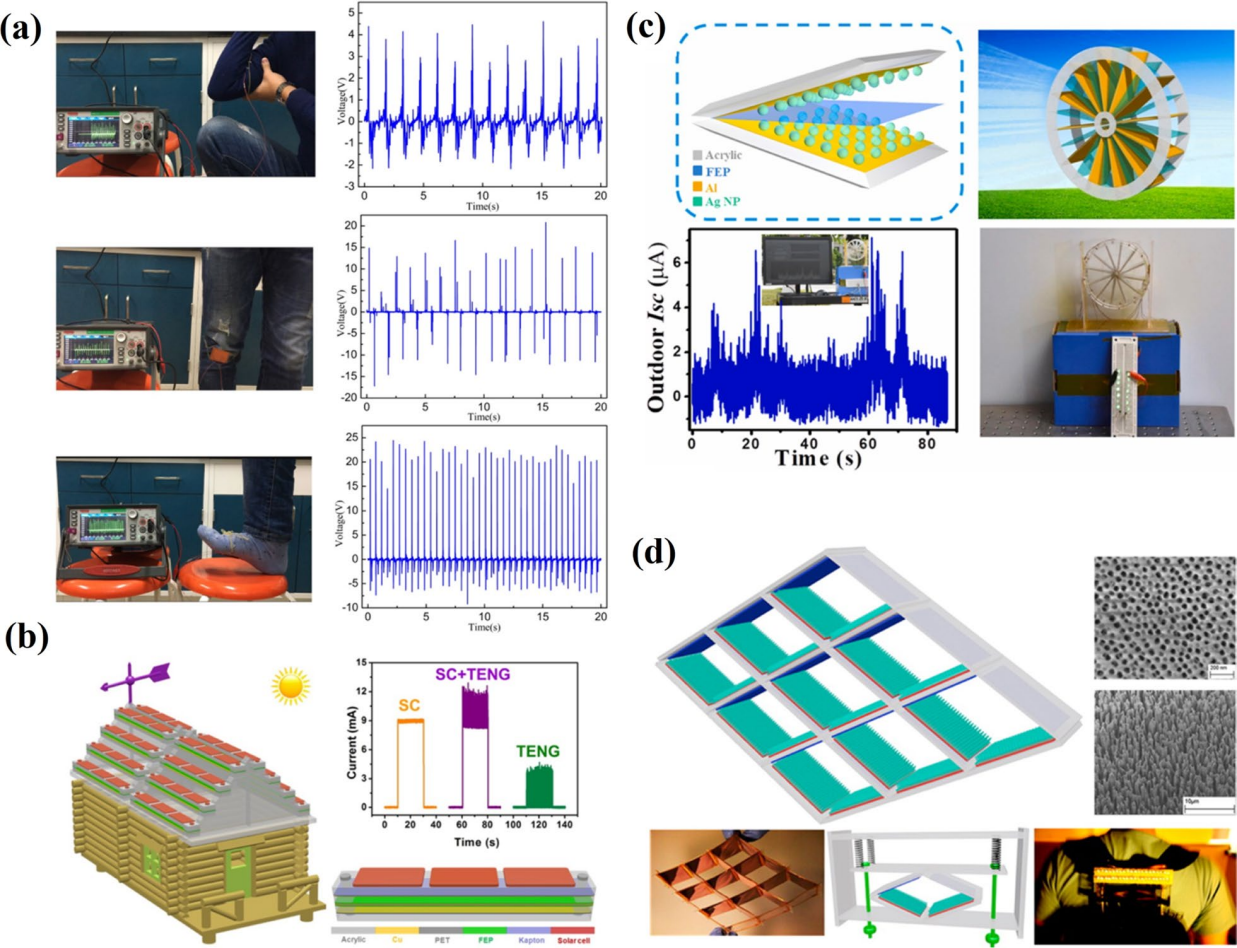
Applications of TENGs for mechanical energy harvesting: **a** a flexible TENG harvesting small mechanical energy from different parts of the body (Reproduced with permission from Ref. [113]); **b** schematic diagram of the integrated hybridized NGs on the roof of a house model (Reproduced with permission from Ref. [114]); **c** TENG for harvesting environmental wind energy (Reproduced with permission from Ref. [115]); **d** a self-powered backpack on the basis of a TENG with integrated rhombic gridding for harvesting the vibration energy from natural human walking (Reproduced with permission from Ref. [116])

Among various ambient energy types, harvesting energy from human-body motion is the most attractive for charging electronic devices and biomedical applications because the human movements have an abundant amount of energy up to several Watts at different body locations. The other natural mechanical energies (vibration, wave and wind energies) have also been studied as a potential energy source for electricity generation. Yan et al. [113] developed TENG based on thermoplastic polymeric nanofiber membranes fabricated through the melt-blending extrusion method to harvest the low-frequency mechanical energy produced by human motion (Fig. 17a). A real application has been demonstrated through hand tapping, when *V*_*oc*_ and *I*_*sc*_ reached up to 340.8 V and 73.7 μA, respectively. Moreover, after modification, the TENG exhibited excellent humidity resistance.

A hybrid NG which can individually/simultaneously harvest the solar and wind energies by applying solar cells and TENG has been demonstrated by Wang et al. [114]. This device is applicable in urban areas to harvest huge amounts of wasted wind energy, as ordinary wind turbine generators require remote areas due to their dimensions and safety issues (Fig. 17b).

Another angle-shaped TENG (AS-TENG) with an enhanced contact area between the triboelectric surfaces to harvest wind energy has also been reported in [115]. The big surface area of the device is due to its design, where two Al layers stacked to form an angle shape and an FEP film positioned in-between share a common side. This part can contact fully and intimately with Al layers to facilitate the effect of the contact electrification and electrostatic induction. The AS-TENG was suggested to harvest weak outdoor wind and act as a direct power source to charge a capacitor or drive electronic devices (Fig. 17c). Based on a TENG with integrated rhombic gridding simultaneously working at both the contact-separation mode and sliding electrification mode, a self-powered backpack has been developed with a vibration-to-electric energy conversion efficiency of up to about 11% (Fig. 17d) [116]. This TENG was viewed as a mobile power source as it produced *V*_*oc*_ up to 428 V and an *I*_*sc*_ of 1.395 mA with a peak power density of 30.7 W/m^2^. A 3D-TENG operating at both the vertical contact-separation mode and the in-plane sliding mode for harvesting random vibrational energy in multiple directions over a wide bandwidth has been developed [117]. The corresponding TENG during out-of-plane and in-plane excitations showed maximum power densities of 1.35 W/m^2^ and 1.45 W/m^2^, respectively. TENG's practical applications have been demonstrated, including harvesting wind or rain-droplet-induced vibrational energy from the national grid transmission lines, natural vibration energy from human walking, and rotation energy from wheel-based vehicles, so making TENG suitable for environmental/infrastructure monitoring and charging portable electronics.

### TENGs as an Active Sensor

**Fig. 18:**
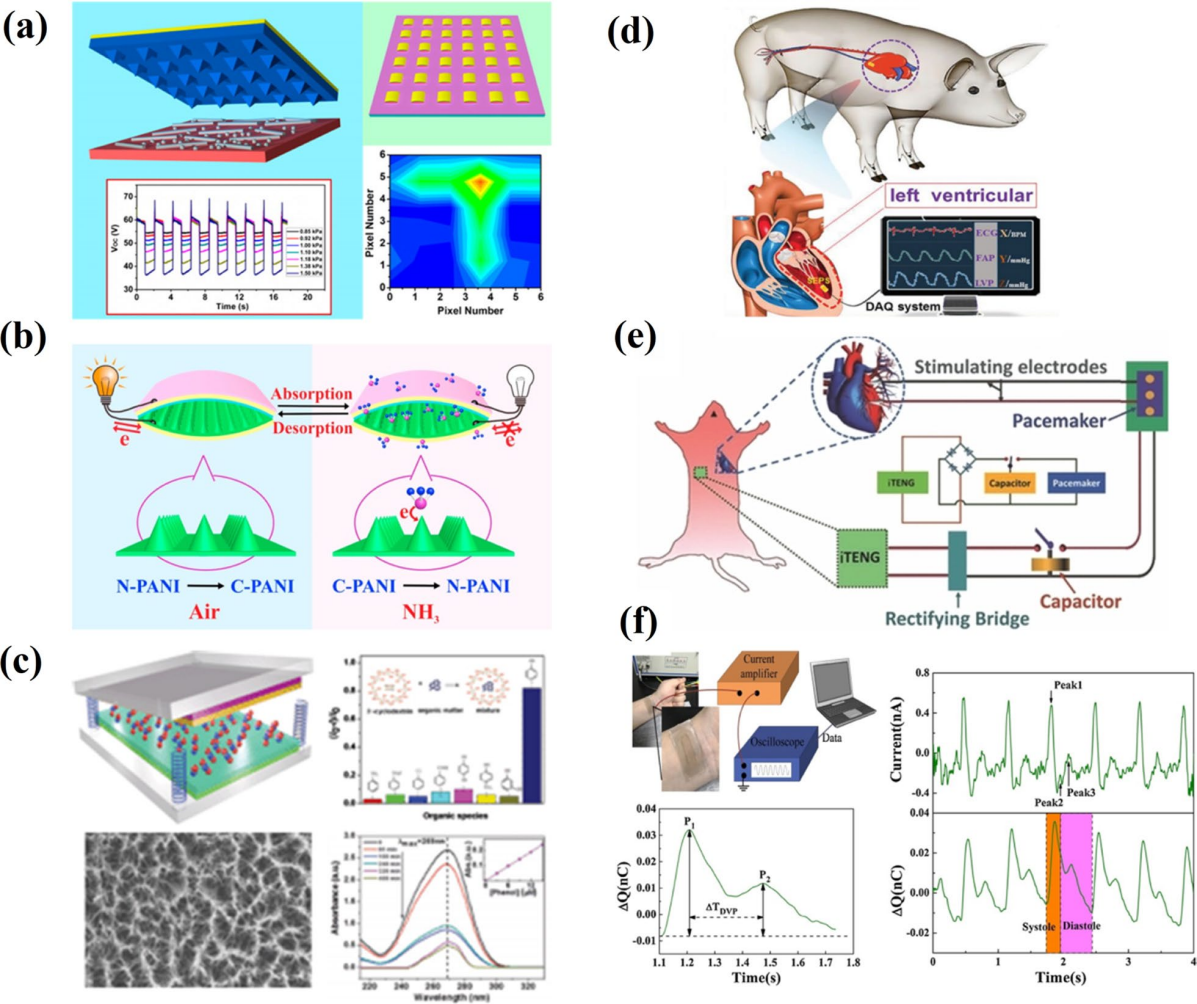
Different applications of TENG: **a** a sensor array for self-powered static and dynamic pressure detection and tactile imaging (Reproduced with permission from Ref. [118]); **b** a self-powered ammonia nanosensor developed from conducting polyaniline nanofibers (Reproduced with permission from Ref. [119]); **c** self-powered detection sensor and electrochemical degradation of phenol utilizing β-cyclodextrin enhanced triboelectrification (Reproduced with permission from Ref. [120]); **d** a self-powered endocardial pressure sensor implanted into a swine’s heart (Reproduced with permission from Ref. [121]); **e** a diagram of an in vivo self-powered prototype of a pacemaker from the breath of a living rat (Reproduced with permission from Ref. [122]); **f** a pulse sensor based on SETENG (Reproduced with permission from Ref. [123])

The quantitative relation between the external inputs and electrical outputs allows TENGs to keep track of ambient changes in real-time applications makes them possible active sensors. The TENG-based active sensors for monitoring pressure, strain, the concentration of chemicals, and humidity have been developed. The triboelectric active sensor (TEAS) that relies on the distinctive pressure response characteristics of *V*_*oc*_ and *I*_*sc*_ and also capable of static and dynamic pressure sensing has been reported in [118]. TEAS have demonstrated high sensitivity (of 0.31/kPa), ultrafast response (< 5 ms) and low detection limit (of 2.1 Pa). Furthermore, tracking and recording the local pressure distribution applied on a device with different spatial profiles has been achieved by integrating multiple TEAS units into a sensor array. That makes a tactile imaging technique applicable in the area of human-electronic interfacing and self-powered systems (Fig. 18a). The possibility of TENGs to be implemented as chemical sensors is related to the change in contact electrification when chemicals are adsorbed on the contact surface and the corresponding dependence of the electrical outputs on the concentration of the chemical substance. A self-powered gas nanosensor TENG based on conducting polyaniline nanofibers (PANI NFs) for detecting ammonia (NH_3_) was suggested by Wang et al. (Fig. 18b) [119]. The corresponding TENG was fabricated from two PANI types, such as conducting emeraldine salt (C-PANI) and non-conducting emeraldine base (N-PANI). The ability of the device to detect NH_3_ is attributed to the transformation of C-PANI to N-PANI through the absorption of NH_3_ on PANI NFs film, which results in the change of the output performance of TENG. Thus, it demonstrated high output performance in the air that gradually deteriorated after being exposed to various NH_3_ concentrations. The property of β-cyclodextrin (β-CD) specifically binds to phenol was applied to fabricate a TENG active sensor capable of detecting and electrochemical degradation of phenol (Fig. 18c) [120]. Here, β-CD was utilized as the phenol detection element and a surface chemical modifier was used to enhance the electrical output performance of TENG. The device had a sensing range which varied from 10 to 100 μM with a sensitivity of 0.01 μ/M, and as the concentration of phenol went up, the electrical output signal decreased. Moreover, the sensor device placed in phenol-containing wastewater, apart from detecting the phenol concentration, purified the wastewater through electrochemical degradation.

A self-powered miniature endocardial pressure sensor (SEPS) based on an implantable TENG (iTENG), fabricated from PTFE film, as the first triboelectric layer with an ultra-thin Au layer electrode at the back, and Al foil simultaneously serving as the second triboelectric layer and electrode, was demonstrated by Liu et al. [121]. This device converted blood flow energy within the heart chambers into an electric output related to endocardial pressure, ventricular fibrillation, and ventricular premature contraction. The corresponding SEPS was implanted into the left ventricle of a male adult Yorkshire pig, and it was revealed that the voltage outputs of SEPS were highly synchronous to the femoral arterial pressure (FAP) (Fig. 18d). Thus, at rest, the output voltage was 80 mV (systolic FAP < 100 mmHg) and increased linearly as FAP increased from 90 to 180 mmHg. Moreover, the device was able to detect cardiac arrhythmias like ventricular fibrillation and ventricular premature contraction.

A flexible iTENG device composed of PDMS, Kapton and Al foil (Fig. 18e), which can harvest the periodic breathing energy in vivo to power a pacemaker prototype for the first time, was demonstrated by Zheng et al. [122]. This device with dimensions 1.2 cm × 1.2 cm × 0.2 cm was implanted into the left chest skin of a rat for breathing sensing in vivo*.* The output values of *V*_*oc*_ and *I*_*sc*_ of the generated electricity were 3.73 V and 0.14 μA, respectively. The energy harvested by the device from breathing was stored in a capacitor and successfully drove a pacemaker prototype to regulate the heart rate of the rat. Another work [123] reported a pulse sensor based on SETENG fabricated from a PET film with an ITO coating layer stacked with a thin PDMS film with a trench structure (Fig. 18f). A bending-sensitive SETENG detected an incident and reflected pulse waves through contact with the wrist and suggested application in medical monitoring.

## Conclusions

TENGs are seen as one of the most promising energy harvesting technologies in the era of IoT. The development of active self-powered sensors is currently booming and TENGs can be potentially used in this vast field. This review outlined the triboelectrification phenomenon and working modes for TENGs and then summarized recent advances in materials selection and development, physical and chemical modification of contacting surfaces for TENGs in order to improve their output performance, moreover, physical hybridization with other energy harvesting technologies to enhance electrical output has also been included. The review ends with the applications of TENGs in various areas of human life. Huge research efforts have been carried out on tribo-materials that enable high-efficiency conversion, particularly, surfaces containing a fluorine functional group that can attract as many electrons as possible from their counterpart. Aspects like applying eco-friendly and biocompatible materials are also important directions.

Moreover, surface structure modification techniques have to be straightforward and efficient in order for TENG devices to be commercialized. Sustainable long-term high power generation is an essential issue for large-scale applications. Here, TENGs working at contact-separation mode usually demonstrate extended stability and working life, and, an approach such as developing durable TENG with rotational structure is a promising way to generate high power. Understanding the working principles and device optimization has to be improved for TENGs because of research is necessary. The latest prevailing suggestion for the mechanism of contact electrification in solid–solid interaction is electron transfer. Research studies in these directions facilitate TENG technology and allow developing autonomous power units to power many personal electronics and self-powered active sensors sustainably.

The existing achievements in this direction make this technology promising for converting mechanical energy into electrical energy to supply power to portable devices. If the above-mentioned problems are overcome, then this technology will become an integral part of human life.

## Data Availability

All data and materials are available without restrictions.

## Abbreviations

2D: Two dimensional
3D: Three dimensional
1L: One-layer
2L: Two-layer
3L: Three-layer
4L: Four-layer
β-CD: β-Cyclodextrin
AAO: Anodic aluminum oxide
AC: Alternating current
AFR: Aniline formaldehyde resin
AgNW: Silver nanowire
APTES: 3-Aminopropyltrimethoxysilane
AS-TENG: Angle-shaped triboelectric nanogenerator
ATRP: Atom-transfer radical polymerization
BC: Benzyl chloride
CA: Cellulose acetate
CF_4_: Tetrafluoride
CNFs: Cellulose nanofibrils
CNTs: Carbon nanotubes
C-PANI: Conducting emeraldine salt
CTAB: Cetyltrimethylammonium bromide
CTS: Chitosan
CVD: Chemical vapor deposition
DP-TENG: Dome-shaped polydimethylsiloxane triboelectric nanogenerator
EMG: Electromagnetic microgenerator
EVA: Ethylene vinyl acetate
FAP: Femoral arterial pressure
FDM: Fused deposition modeling
FEC: Fluoroethylene carbonate
FEP: Fluorinated ethylene propylene
FOM: Figure of Merit
FOMm: Material figure of merit
FOMp: Performance figure of merit
FOMs: Structural figure of merit
FOTS: (1H,1H,2H,2H-perfluorooctyl)silane
FSTENG: Free standing triboelectric nanogenerator
GO: Graphene oxide
HD-MN-TENG: High density microneedles triboelectric nanogenerator
H-P/TENG: Hybrid piezoelectric-triboelectric nanogenerator
HTMS: Hexyltrimethoxysilane
IBTENG: Infusible biomass-based triboelectric nanogenerator
IoT: Internet-of-Things
I_sc_: Short-circuit current
ITO: Indium tin oxide
Jsc: Short circuit current density
LD-MN-TENG: Low density microneedles triboelectric nanogenerator
LED: Light-emitting diode
LSTENG: Lateral sliding triboelectric nanogenerator
LTV: Low-temperature vulcanized
MF: Melamine formaldehyde
M_w_: Molecular weight
MWCNTs: Multi-wall carbon nanotubes
NaOH: Sodium hydroxide
NFs: Nanofibers
NGs: Nanogenerators
N-PANI: Non-conducting emeraldine base
NR: Natural rubber
NWs: Nanowires
OL-MN: Overlapped microneedles
PA: Polyamide
PANI: Polyaniline
PDMS: Polydimethylsiloxane
PE: Polyethylene
PEG: Polyethylene glycol
PEI: Polyethyleneimine
PEDOT:PSS: Poly(3,4-ethylenedioxythiophene):poly(styrene sulfonate)
PENG: Piezoelectric nanogenerator
PEO: Poly(ethylene oxide)
PET: Polyethylene terephthalate
PHVV: Poly(3-hydroxybutyrate-co-3-hydroxyvalerate)
PLL: Poly-l-lysine
PMMA: Poly(methyl methacrylate)
PP: Polypropelene
PP-TENG: Pillar-shaped polydimethylsiloxane triboelectric nanogenerator
PS: Polystyrene
PtBA: Poly(tert-butylacrylate)
PVDF: Polyvinylidene fluoride
PPy: Polypyrrole
PTFE: Polytetrafluoroethylene
PUA: Polyurethane acrylate
PVDF-TrFE: Polyvinylidene fluoride-trifluoroethylene
RAFT: Reversible addition-fragmentation chain-transfer
rGO: Reduced graphene oxide
SAM: Self-assembled monolayer
SEM: Scanning electron microscopy
SEPS: Self-powered endocardial pressure sensor
SETENG: Single electrode triboelectric nanogenerator
TEAS: Triboelectric active sensor
TENG: Triboelectric nanogenerator
TF-TENG: Transparent flexible triboelectric nanogenerator
TPU: Thermoplastic polyurethane
UVO: Ultraviolet-ozone
VCSTENG: Vertical contact-separation triboelectric nanogenerator
V_oc_: Open-circuit voltage
WRP: Wasted rubber powder
